# Emerging Strategies for Revascularization: Use of Cell-Derived Extracellular Vesicles and Artificial Nanovesicles in Critical Limb Ischemia

**DOI:** 10.3390/bioengineering12010092

**Published:** 2025-01-20

**Authors:** Vijay Murali Ravi Mythili, Ramya Lakshmi Rajendran, Raksa Arun, Vasanth Kanth Thasma Loganathbabu, Danyal Reyaz, ArulJothi Kandasamy Nagarajan, Byeong-Cheol Ahn, Prakash Gangadaran

**Affiliations:** 1 Integrative Genetics and Molecular Oncology Group, Department of Genetic Engineering, College of Engineering and Technology, SRM Institute of Science and Technology, Chengalpattu 603203, Tamil Nadu, India; rmvijaymurali02@gmail.com (V.M.R.M.); raksaarun06@gmail.com (R.A.); vasanthkanthofficial@gmail.com (V.K.T.L.); reyazjunior@gmail.com (D.R.); aruljotn@srmist.edu.in (A.K.N.); 2 Department of Nuclear Medicine, School of Medicine, Kyungpook National University, Daegu 41944, Republic of Korea; ramyag@knu.ac.kr; 3 BK21 FOUR KNU Convergence Educational Program of Biomedical Sciences for Creative Future Talents, Department of Biomedical Sciences, School of Medicine, Kyungpook National University, Daegu 41944, Republic of Korea; 4 Cardiovascular Research Institute, Kyungpook National University, Daegu 41944, Republic of Korea; 5 Department of Nuclear Medicine, Kyungpook National University Hospital, Daegu 41944, Republic of Korea

**Keywords:** critical limb ischemia, extracellular vesicles, nanovesicles, revascularization, regenerative medicine

## Abstract

Critical limb ischemia (CLI) poses a substantial and intricate challenge in vascular medicine, necessitating the development of innovative therapeutic strategies to address its multifaceted pathophysiology. Conventional revascularization approaches often fail to adequately address the complexity of CLI, necessitating the identification of alternative methodologies. This review explores uncharted territory beyond traditional therapies, focusing on the potential of two distinct yet interrelated entities: cell-derived extracellular vesicles (EVs) and artificial nanovesicles. Cell-derived EVs are small membranous structures naturally released by cells, and artificial nanovesicles are artificially engineered nanosized vesicles. Both these vesicles represent promising avenues for therapeutic intervention. They act as carriers of bioactive cargo, including proteins, nucleic acids, and lipids, that can modulate intricate cellular responses associated with ischemic tissue repair and angiogenesis. This review also assesses the evolving landscape of CLI revascularization through the unique perspective of cell-derived EVs and artificial nanovesicles. The review spans the spectrum from early preclinical investigations to the latest translational advancements, providing a comprehensive overview of the current state of research in this emerging field. These groundbreaking vesicle therapies hold immense potential for revolutionizing CLI treatment paradigms.

## 1. Introduction

Peripheral arterial disease (PAD) is a chronic disease of the vascular system characterized by lower levels of circulation to the lower extremities of the body. Critical limb ischemia (CLI) is by far considered the most severe form of PAD. It is characterized by the presence of ischemic rest pain, ulcer, or gangrene that is attributed to arterial occlusive disease [1]. Moreover, it is the final stage of lower extremity arterial disease (LEAD), which is estimated to affect millions of people worldwide. LEAD is the endpoint in the equilibrium between the metabolic supply in the system and the demand of the lower extremities. The most common causes of PAD are atherosclerosis and other potential risk factors, such as smoking, hypertension, hypercholesterolemia, diabetes mellitus, and obesity. The severity of the condition is exacerbated by various other conditions, such as myocardial infarction, stroke, and loss of limbs. In some cases, it is also reported to cause death [2,3]. Globally, nearly 200 million people are affected by CLI, with most patients experiencing a sixfold increase in the rate of cardiovascular events. In addition to atherosclerosis, thromboembolism, vasculitis, trauma, and adventitial cystic disease are reported to be associated with the onset of CLI, thereby broadening the range of pathophysiological features [4,5]. Treatment options, such as wound healing and limb amputation, prevent the progression of cardiovascular events in patients. Revascularization, the most popular technique, has been shown to provide adequate amounts of oxygen to the affected areas in at least half of the patients with CLI. The mode of revascularization could be either surgical or endovascular, depending on the severity of the condition [6]. CLI can be diagnosed by the presence of low ankle or toe pressure or reduced levels of transcutaneous oxygen (TcO_2_), which are used to predict foot perfusion and, consequently, wound healing. In patients with CLI, ankle pressures range from less than 40 mmHg to 70 mmHg, while toe pressures range from 30 mmHg to 50 mmHg. Moreover, TcCO_2_ levels range from less than 20 mmHg to 40 mmHg. The simplest method for diagnosis is the ankle–brachial index (ABI), which is the ratio of the highest ankle pressure in a particular limb to the highest brachial pressure. An ABI value of less than 0.9 is an indicator of PAD, while an ABI value of 0.4 is indicative of CLI [7,8]. Various imaging techniques, such as computed tomography angiography and magnetic resonance angiography, have been used to visualize the lower extremity regions before providing a definite treatment plan for patients [9]. Various classification systems, including Fontaine, Rutherford, and Wagner classifications, are summarized in Table 1. These classification systems serve as frameworks for classifying the severity of CLI according to various clinical characteristics, including ischemic symptoms, ulceration, and the presence or amount of gangrene. They help medical professionals standardize patient diagnosis and treatment planning, enabling them to evaluate the disease course and successfully customize therapeutic approaches. They also provide a comparative overview of the different clinical manifestations of CLI, further facilitating clinical judgment and research to treat this crippling illness.

Revascularization is the most preferred first-line treatment for CLI. It can be performed either through the bypass method or through the endovascular method. The main objective of this technique is to prevent tissue loss by establishing circulation back to the foot. In the case of aortoiliac occlusive disease, anatomic and extra-anatomic bypasses can be used by implementing prosthetic grafts. The chances of performing revascularization on any patient depend on various factors, such as the patient’s age, severity of CLI, vascular anatomy, and availability of vein grafts specific to the particular patient [4]. The aortobifemoral bypass technique is more commonly used than iliofemoral and thoracofemoral techniques for aortoiliac revascularization [15]. A randomized control trial by Bradbury et al. [15], known as the Bypass versus Angioplasty in Severe Ischemia of the Leg (BASIL) trial, included 452 patients and focused on the outcomes of patients who underwent bypass surgery versus those who underwent angioplasty for CLI due to infrainguinal disease. The results revealed no significant differences in the primary endpoint of amputation-free survival between both groups. A limitation of this trial was the sole use of angioplasty, considering the endpoint of amputation-free survival. Another limitation was the inefficiency in studying the anatomical pattern of the disease because of the effect of revascularization [15,16]. The revascularization process can also be inefficient in certain patients with severe atherosclerosis because of the extreme pattern of arterial disease along with long calcified blockages and below-the-knee arterial diseases [17]. Considering the failure and inefficiency of pre-existing revascularization techniques, a novel extra-anatomic femoropopliteal bypass technique was developed for amputation in patients with CLI and a history of conventional revascularization failure. However, the outcome of this procedure was quite unfavorable because of its higher hemorrhagic risk than conventional procedures. In particular, one patient died of cardiogenic shock, while one patient experienced an acute coronary syndrome associated with iliopsoas bleeding [18]. Yannoutsos et al. studied the outcomes of 315 patients who underwent revascularization surgery. In their study, 3.8% of the patients died after a month of revascularization. The mortality rate increased to approximately 25.4% after a year [19]. In addition to limb loss, the increase in mortality rates among patients with CLI has become a global concern. In another study, Agarwal et al. identified that major amputations were a leading cause of in-hospital mortality among patients owing to the major risks involved in the amputation process [20]. Cardiovascular events play an important role in the management of CLI. Patients with CLI are more prone to cardiovascular events, such as myocardial infarction (MI), stroke, and complications leading to death. Patients with CLI who experience cardiovascular events require appropriate medical care and treatments that can determine their treatment outcome. Therefore, it is necessary to handle cardiovascular events and to simultaneously maximize patient output and minimize mortality rates. This can be accomplished by minimizing risk factors, such as hypertension, hyperlipidemia, and diabetes [1,5]. Through the synchronization of care and the incorporation of treatment strategies, healthcare teams can ensure that patients promptly receive therapies for both cardiovascular events and CLI [7].

## 2. Role of Extracellular Vesicles in CLI Revascularization

### 2.1. Rationale for Use of EVs and Nanovesicles in Revascularization

EVs are membrane-bound organelles produced by cells in the extracellular region. They play a role in intercellular communication and transport [21,22]. The therapeutic effect of EVs on CLI has also been studied. In particular, stem cell-derived EVs have shown potential for enhancing neovascularization, thereby aiding in tissue regeneration and recovery [23,24,25]. Xing et al. employed a dual-pathway activation strategy involving EVs loaded with vascular endothelial growth factor (VEGF) and transcription factor EB (TFEB) in combination with a thermoresponsive hydrogel for delivery. In their study, modified EVs were prepared by genetically modifying parent cells to express VEGF and TFEB in order to improve their bioactivity and therapeutic potential. VEGF and TFEB were chosen because they enhance the bioactivity of EVs; angiogenesis is crucially dependent on VEGF, while autophagy is regulated by TFEB. This combination of EVs with hydrogel significantly improved neovascularization, reduced muscle injury, and restored limb function in animal models. Moreover, mechanistic experiments revealed that the therapeutic impacts were mediated through the activation of the VEGF/VEGFR pathway and the autophagy–lysosomal pathway. The use of a thermoresponsive hydrogel for the delivery of EVs has been proven to be effective in prolonging EV retention in vivo and releasing EVs in response to the severity of CLI, thus providing a non-invasive treatment strategy for patients with CLI [26,27,28]. In another study, Mendhe et al. assessed the use of lyophilized EVs from adipose stem cells in a mouse hindlimb ischemia model to reduce tissue damage associated with ischemia–reperfusion injury. The results revealed that EVs increased reperfusion and the expression of the anti-inflammatory factor annexin A1 in the skeletal muscles. However, the increase in reperfusion significantly decreased the levels of muscle structural proteins, such as dystrophin, plectin, and obscurin. Furthermore, an increase in inflammatory cytokines, such as TNF-α and IL-6, was observed upon EV treatment, with the serum TNF-α level positively correlating with reperfusion levels [29,30,31].

### 2.2. Biogenesis and Classification of EVs

**Figure 1:**
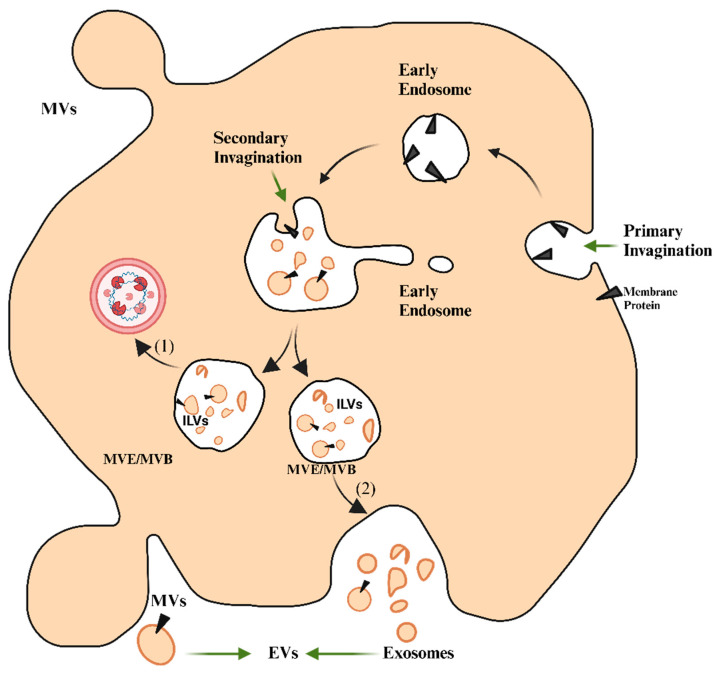
Processes involved in the production of extracellular vesicles (exosomes and microvesicles). As the process continues, early endosomes develop into late endosomes or multivesicular bodies (MVBs). During maturation, the membrane of MVBs undergoes invagination, resulting in the formation of intraluminal vesicles (ILVs) within MVBs. Ultimately, some of the MVBs were recylted by lysosome (refer to 1) and others MVBs merge with the plasma membrane (refer to 2), liberating ILVs as exosomes into the surrounding extracellular space. This merging process is mediated by the coordinated action of the cytoskeleton, Rab family GTPases (such as Rab27a and Rab27b), SNARE proteins, and tethering complexes, which facilitate the docking and fusion of MVBs with the plasma membrane. On the other hand, microvesicles are created through direct outward budding and shedding of the plasma membrane, resulting in their release in the extracellular environment [40].

EVs are divided into medium/large EVs (>200 nm) and small EVs (sEVs) (<200 nm) based on their physical properties [32]. They can be classified based on their size, biogenesis, and cargo content. Based on their size, EVs are further categorized into subtypes, such as apoptotic bodies, exosomes, and microvesicles. Exosomes are small in size and range from 30 to 150 nm in diameter. They originate from the endosomal system, primarily from multivesicular bodies (MVBs) in the form of intraluminal vesicles (ILVs) that are released into the extracellular space in the form of exosomes. Exosome biogenesis occurs in two steps. The first step involves the reorganization of the endosomal membrane to increase the specificity to tetraspanins [33]. Figure 1 provides a detailed representation of the biogenesis and secretion of EVs, depicting the intricate biological processes involved. It demonstrates how MVBs produce ILVs, which then fuse with the plasma membrane to release exosomes into the extracellular space. The figure also reveals the direct outward budding of microvesicles from the plasma membrane, which is determined by complex molecular interactions encompassing pathways involved in cell signaling and cytoskeletal structures. The key tetraspanins involved in this process are CD9 and CD63, which are thought to play an important role in exosome formation. In the second step, the endosomal sorting complexes required for transport (ESCRTs) are recruited to the ILV formation site where the exosomes are formed. Four ESCRTs, namely ESCRTs 0, I, II, and III, are involved in this process. ESCRT 0 recognizes the proteins ubiquitinated on the endosomal membrane, while ESCRTs I and II are recruited to the cytosolic fraction of early endosomes through various stimuli. Intraluminal membrane budding is initiated and driven by the action of ESCRTs I and II, while ESCRT III terminates the process in association with other proteins, such as ALIX and TSG101 [34]. On the other hand, microvesicles are comparatively larger in size and range from 100 to 1000 nm in diameter. The formation of microvesicles occurs via outward budding and fission of the plasma membrane, because of which the vesicles disperse into the extracellular environment. This process begins when ADP-ribosylation factor 6 (ARF6) initiates a cascade for the release of microvesicles by activating phospholipase D (PLD) and recruiting extracellular signal-regulated kinase (ERK) to the plasma membrane, and further phosphorylates and activates the myosin light chain kinase (MLCK). Subsequently, microvesicles are released through the contraction of actin–myosin machinery [35]. This size- and biogenesis-based classification of EVs highlights the distinguishing features of exosomes and microvesicles, thereby providing insights into the mechanism underlying their biogenesis and origin at the cellular level, and their roles in various processes, such as intercellular signaling and disease progression [36]. Stem cells can differentiate into cells of any kind, thereby aiding in the body’s repair mechanism [37]. The two main types of stem cells are embryonic stem cells (ESCs) and adult stem cells, such as mesenchymal stem cells (MSCs). ESCs are pluripotent and can differentiate into any cell type. ESC-derived EVs are involved in the migration of trophoblasts, implantation of blastocysts, and maintenance of stem cell characteristics through various molecules, such as fibronectin and heat shock protein (HSP)90 [38]. MSC-derived EVs (MSC-EVs) carry specific markers, such as CD105, CD90, and CD73, which can influence the behavior of other cells by promoting cell proliferation, preventing the apoptosis of hematopoietic stem cells, and modulating immune responses [39].

### 2.3. Cellular Sources of Therapeutic EVs

The sources of therapeutic EVs include a wide range of live cells that release nanovesicles in the extracellular space. The study included four main steps: tissue/cellular RNA-seq data processing, construction and optimization of signature matrices, model selection and evaluation, and exploration of the atlas of EV origins from healthy or diseased samples. Two signature matrices were developed—one for blood cells and another for tissue types—using specific genes identified through a tissue-specific scoring strategy. Six deconvolution algorithms were tested, with the support vector regression (SVR) method, selected as the core algorithm for EV origin because of its robustness and accuracy. Performance evaluation of the models revealed that the SVR method exhibited higher concordance and lower differences between known and estimated tissue/cell type proportions than the other methods. The EV origin approach successfully depicted the contribution of tissue/cellular sources of circulating EVs in healthy individuals and patients with diseases, particularly highlighting the significance of the liver fraction in diagnosing hepatic disorders, such as hepatocellular carcinoma.

### 2.4. Biophysical Experimental Approaches for Elucidating Exosome Release and Drug Loading Mechanisms

Biophysical methods are indispensable for understanding the mechanisms involved in exosome release and drug loading and unraveling their structural and functional dynamics. Dynamic light scattering (DLS) and nanoparticle tracking analysis (NTA) are often used to monitor changes in exosome size and concentration upon drug loading. Cryo-EM enables high-resolution imaging of exosomal membranes, thereby unraveling structural alterations post loading. Fluorescence spectroscopy using fluorescence resonance energy transfer (FRET) facilitates the real-time monitoring of the incorporation and release of drugs, while surface plasmon resonance helps measure drug molecule interactions with exosomal membranes. Atomic force microscopy provides topographical and mechanical information regarding exosomal membrane stability and permeability by determining changes after drug incorporation. In addition, mass spectrometry (MS) assesses the molecular composition of exosomes, providing insights into proteomic and lipidomic alterations upon drug loading or inflammatory exposure. Isothermal titration calorimetry (ITC) further supports this by unraveling the thermodynamics underlying drug binding to exosomal membranes [41,42].

### 2.5. Mechanisms of EV-Mediated Revascularization

EVs play an important role in mediating revascularization through various processes by transferring biomolecules, such as microRNAs (miRNAs), into endothelial cells and inducing angiogenesis [43,44,45,46]. EVs are involved in the delivery of proangiogenic miRNAs to endothelial cells, which can stimulate vascular growth and maturation and thereby promote neovascularization post myocardial infarction. A study conducted by Berger et al. revealed that EVs released after remote ischemic conditioning can be transferred across species and confer protection against hypoxia, highlighting their role in mediating cardioprotection. These EVs accumulate in the injured myocardium, thereby reducing infarct size and facilitating revascularization processes [47,48]. Vicencio et al. reported the cardioprotective capability of EVs in conferring protection against ischemia–reperfusion injury. They identified EVs as crucial mediators of the cardioprotective signal induced by remote ischemic conditioning. Specific proteins carried by EVs, including the classic cardioprotective HSP70, were also identified as mediating substances [49]. HSP70 plays a protective role in cellular stress responses and can promote cell survival during ischemic events. The presence of HSP70 in EVs suggests that these vesicles transfer not only miRNAs but also essential proteins that contribute to cardioprotective effects. This finding underscores the multifaceted nature of EV-mediated cardioprotection, wherein both miRNAs and proteins (such as HSP70) work synergistically to promote cell survival and tissue protection during ischemia–reperfusion injury [49].

### 2.6. Preclinical Evidence Supporting EV Therapy in CLI

The use of EVs for the treatment of CLI is promising because it enhances the therapeutic outcomes. Shen et al. assessed the use of sEVs derived from hypoxic human umbilical vein endothelial cells (HUVECs) to enhance the therapeutic potential of adipose-derived MSCs (AD-MSCs) in CLI [50,51,52,53]. Hypoxic sEVs (hsEVs) have a different composition from normal sEVs. This increases the level of miR-486-5p, which downregulates PTEN via direct targeting, thereby activating the AKT/mTOR/HIF-1α pathway in AD-MSCs. This activation increases the survival and proangiogenic ability of AD-MSCs, resulting in enhanced cell engraftment, angiogenesis, and tissue repair, as noted in a hindlimb ischemia model in a previous study. In that study, hsEVs were found to improve the resistance of AD-MSCs to reactive oxygen species (ROS) and enhance their proangiogenic capacity through miR-486-5p-mediated PTEN repression, ultimately enhancing the therapeutic efficacy of AD-MSCs in CLI [54].

## 3. Artificial Nanovesicles: Design and Function

### 3.1. Engineering Strategies for Nanovesicle Production

Various strategies have been employed to increase the efficiency of nanovesicles. The genetic manipulation of exosome biogenesis pathways and pre-treatment of parent cells are potential techniques used to enhance yield. Techniques like genetic engineering involve the introduction of foreign genes into the recipient cells via an in vitro recombination process that can enhance exosome biogenesis and release pathways [55]. Pre-treatment of parent cells by subjecting them to hypoxic conditions, cytokines, thrombin, and adiponectin can increase the yield of exosomes. These conditions increase exosome production, thereby causing an exponential increase in exosome release [56]. Changes in the outer structure of exosomes can enhance nanovesicle production. This can be achieved via alterations in the conditions in which they are produced. In the presence of nitric oxide (NO)-releasing polymers, exosomes from MSCs were found to increase the angiogenic properties of vesicles [57,58]. Shi et al. proposed a different strategy involving the use of polyethylene glycol (PEG)-modified exosomes. In their study, copper-64-radiolabeled PEG exosomes were monitored using positron emission tomography. They found that the exosomes with PEG exhibited higher drug delivery quality than those without PEG [59].

### 3.2. Cargo Loading and Modification of Nanovesicles

Cargo loading of EVs, particularly of exosomes, is a crucial aspect of drug delivery. Various methods have been explored to efficiently load cargo into these nanovesicles. Different strategies, such as rapid squeezing through nanofluidic channels, have been developed to load exogenous cargo into EVs by temporarily generating nanopores on the membrane. Islam et al. developed a systemic algorithm to predict drug loading during EV squeezing using coarse-grain (CG) molecular dynamic simulations coupled with fluid dynamics. They analyzed the effects of various parameters, such as EV size, flow velocity, and channel dimensions, on pore formation and drug loading efficiency, providing a phase diagram for nanochannel design [60]. Other cargo-loading approaches, such as electroporation, sonication, and incubation, have been adopted to increase the cargo loading of EVs. The electroporation process involves the application of an electric field from an external source right after the exosomal membrane. In the presence of a high voltage supplied for a short duration, the lipid layer ruptures and causes a temporary state of membrane permeability, allowing other small molecules to pass through the membrane. Zhu et al. successfully loaded doxorubicin into exosomes derived from lens epithelial cells via electroporation; this led to an enhanced uptake of the exosomes by the lens epithelial cells [61,62]. The sonication method of cargo loading follows the same procedure by disrupting the membrane of exosomes, thereby facilitating drug loading into the exosomes. Sonication employs mechanical shear forces through ultrasound waves, temporarily disrupting the lipid bilayer of exosomes. This disruption creates transient pores or destabilizes the membrane structure, allowing the drug molecules in the surrounding solution to diffuse into the exosomal lumen. Once sonication ceases, the membrane reseals, encapsulating the drug molecules within the exosomes. Yerneni et al. successfully performed sequential loading of albumin and curcumin into exosomes through the sonication process, which resulted in the formation of carrier exosomes that possessed greater stability and anti-inflammatory properties [63]. The loading of small molecules into exosomes via the incubation method is one of the most common ways to increase the cargo-loading capacity of exosomes. The incubation method relies on the passive diffusion of small molecules across the lipid bilayer of the exosomal membrane. This process is primarily driven by the concentration gradient of small molecules in the surrounding medium. The efficiency of loading depends on the physicochemical properties of the molecules, such as their polarity, hydrophobicity, and molecular size. Non-polar or hydrophobic molecules generally diffuse more readily into the lipid-rich environment of the exosomal membrane and lumen, whereas polar or hydrophilic molecules may face greater resistance. The incubation conditions, including temperature, duration, and pH, also play a key role in optimizing the loading efficiency. Wang et al. modified exosomes with RGD peptide and incubated them with doxorubicin, which resulted in drug loading into the exosomes. The exosomes labeled with ^131^I efficiently targeted tumor cells because of the presence of RGD peptide, which serves as a targeting molecule [64]. EV modifications play a pivotal role in increasing drug delivery [65]. Various strategies, such as the use of cell membrane nanovesicles, cell-derived nanovesicles, and lipid nanovesicle structures (e.g., liposomes), have been developed for efficient drug delivery mechanisms.

### 3.3. Targeting and Delivery Mechanisms

**Figure 2:**
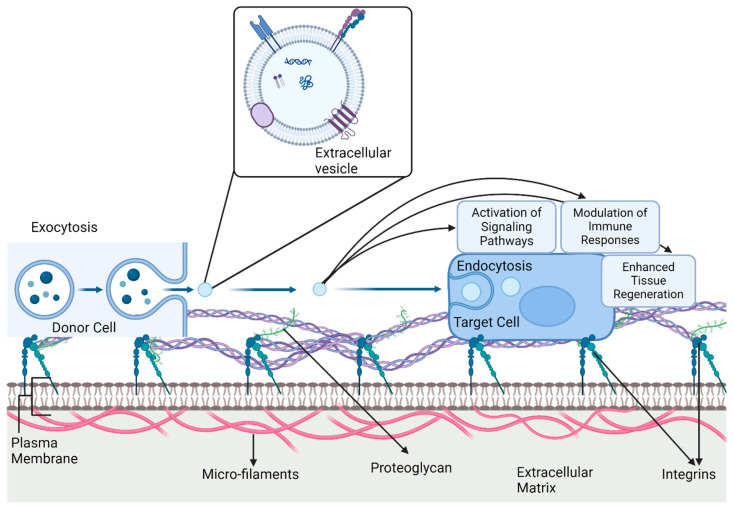
The series of intracellular events that occur when recipient cells take up extracellular vesicles (EVs) through endocytosis. When EVs merge with the recipient cell membrane, their contents are released into the cytoplasm, resulting in the activation of different signaling pathways. This process alters immune responses by transporting bioactive molecules, such as cytokines and microRNAs, which can either enhance or suppress immune cell activity. Moreover, the release of growth factors and other regenerative molecules from EVs improves tissue repair and regeneration. The figure provides a visual representation of the intricate relationship between EVs and target cells, emphasizing the intricate molecular processes involved in signal transduction, immune modulation, and tissue regeneration, Created with BioRender.com.

Nanovesicles exhibit targeted drug delivery mechanisms and possess inherent homing abilities because of the presence of enriched membranes with specific receptors or ligands that interact with target cells. Various modifications, such as surface alterations and source cell changes, can enhance their targeting specificity [66]. For instance, apoptotic body-mimetic nanovesicles that are derived from apoptotic fibroblasts and conjugated with dextran and ischemic cardiac homing peptides can actively target the ischemic myocardium and polarize macrophages to exhibit anti-inflammatory effects [67]. Additionally, artificial nanovesicles encapsulating therapeutic agents can protect cargo from degradation, enabling prolonged efficacy against pathogens, such as *Botrytis cinerea*. These findings highlight the potential therapeutic applications of nanovesicles in precision medicine for targeted and efficient drug delivery [68]. Targeting by nanovesicles consists of two types: passive and active. Passive targeting relies on the enhanced permeability and retention effect, wherein nanovesicles accumulate in tumor tissues because of their leaky vasculature and impaired lymphatic drainage. On the other hand, active targeting involves the modification of nanovesicles with ligands or antibodies that specifically bind to receptors on target cells, enhancing their uptake and efficacy [66]. Advancements in nanoengineering techniques have enabled the modification of EVs, such as the alteration of the source cell or surface of EVs, to improve specific targeting. Overall, the understanding and optimization of these targeting mechanisms are crucial for enhancing the precision and effectiveness of nanovesicle drug delivery systems in various diseases [69]. The internalization and delivery of nanovesicles predominantly occur via receptor-mediated endocytosis, resulting in the accumulation of nanovesicles inside the lysosomes [70]. These nanovesicles play a crucial role in drug delivery systems, enhancing bioavailability and therapeutic efficacy by solubilizing, encapsulating, and stabilizing active ingredients for oral administration [71]. Ou et al. assessed the cellular uptake mechanisms and immunogenicity profile of novel bio-hybrid nanovesicles (nCVTs), developed by fusing the membranes of U937 monocytes with synthetic lipids. They found that the nCVTs exhibited approximately 40-fold higher cellular internalization than liposomes within the first hour, primarily through receptor-mediated processes, such as clathrin- and caveolae-mediated endocytosis. Furthermore, the nCVTs exhibited low immunostimulatory potential both in vitro and in vivo, as indicated by the levels of IL-1α, IL-6, and TNF-α cytokines [72]. Figure 2 presents the processes associated with the cellular uptake of EVs, including intracellular mechanisms. EVs engage their recipient cells to transfer inducted loads, which may comprise proteins, lipids, cytokines, and nucleic acids, to the cytoplasm. The signaling pathways have essential intercellular controls for most biological processes, including angiogenesis, immunoregulation, and tissue repair. The figure provides a comprehensive view of how EV-mediated communication modifies cellular responses to improve treatment outcomes in ischemic circumstances, such as CLI.

## 4. Comparative Analysis: EVs Versus Artificial Nanovesicles

### 4.1. Efficacy and Safety Profiles

The surface charge, shape, size, and composition of artificial nanovesicles can influence their toxicity by affecting cellular uptake and biological responses. Preclinical studies play a crucial role in evaluating the safety, efficacy, and pharmacokinetics of artificial nanovesicles before advancing to clinical trials [73]. Preclinical studies have indicated that artificial nanovesicles can effectively target and deliver therapeutic cargo to specific targets. Further optimization strategies can enhance the safety profile of artificial nanovesicles, including enhancement of the formulation, optimization of therapeutic cargo loading methods, and surface modifications [74].

### 4.2. Mechanistic Differences

The mechanistic aspects of EVs and artificial nanovesicles include lipid composition, protein content, uptake mechanisms, and functional receptor transfer [75]. The lipid composition of EVs can vary based on the cell type and differentiation stage. EVs have specific lipid compositions, such as sphingomyelin and gangliosides content. Changes in the lipid composition during cellular differentiation can influence the function of EVs [76]. The proteins carried by EVs are important for their functions, mediating interactions with target cells and initiating intracellular cascades [77]. Cell signaling, immune responses, and tissue regeneration processes are influenced by the biological effects of EVs on recipient cells, which can be determined by the protein cargo of EVs. EV uptake by recipient cells can occur through different mechanisms, including phagocytosis and micropinocytosis (a form of endocytosis) [78]. These are influenced by the characteristics of both EVs and recipient cells. The uptake process can also be impacted by the fluidity of cellular membranes and the presence of specific receptors on EVs and target cells [79]. EVs can transfer functional receptors to target cells, causing changes in the phenotype and behavior of the cells. The cellular responses and signaling pathways can be modulated by the transfer of receptors. This affects various physiological and pathological processes [80]. Artificial nanovesicles are designed in a way that enables them to actively target disease sites, respond to signals, and provide treatment feedback [81]. To achieve enhanced targeting, these nanovesicles are engineered by modifying their surface with ligands or antibodies that have a high binding affinity with the receptors or antigens present on the target cells. Surface modifications are engineered based on the target cells or tissues to enable precise drug delivery [68]. Artificial nanovesicles can react to both internal and external signals, thereby providing a stimulus-responsive drug release system that can be activated by various triggers, such as ultrasound or light, to improve control over drug delivery. By integrating feedback modules, nanovesicles can monitor the treatment response of the host, enabling real-time assessment of drug efficacy and disease progression [82]. This feedback allows artificial nanovesicles to adapt their therapeutic cargo release or targeting strategies based on the host’s response, thereby optimizing the outcome [83].

### 4.3. Potential Synergies in Combination Therapies

EVs exhibit intrinsic tissue-homing capabilities because of their surface proteins and lipids, which allow them to effectively deliver therapeutic cargo to specific cells or tissues. This can enhance overall treatment efficacy [84]. Small molecules often exert their therapeutic effects through distinct mechanisms. By combining small molecules with EV-based drug delivery, complementary actions can be achieved. This serves as a more comprehensive treatment approach that can target multiple pathways involving the disease [85]. This combinatorial approach maximizes the therapeutic potential of each component, resulting in increased efficacy. A combination of MSC-EVs and other regenerative therapeutic agents, such as scaffolds or growth factors, can amplify tissue repair and regeneration, thereby providing a synergistic strategy for tissue healing [86]. Artificial nanovesicles provide a platform for combination therapies because of their unique properties and specificity [87]. They can be integrated with different therapeutic agents, such as miRNAs, which enables a combination of treatments with a single delivery system [88].

## 5. Preclinical Models and Studies

### 5.1. Animal Models of CLI for EV and Nanovesicle Research

Hindlimb ischemia induced by the surgical ligation of the femoral artery in female BALB/c mice replicates the pathophysiological conditions observed in human CLI [89,90]. This method creates a model that closely mimics the arterial blockages observed in human CLI, characterized by considerable blood flow reduction, tissue ischemia, and necrosis. The use of this model allows researchers to study the effects of ischemia on limb perfusion, tissue damage, and therapeutic interventions systematically [91]. The relevance of this model to human CLI is significant; this similarity ensures that findings from the mouse model, particularly those regarding the progression of tissue damage and the potential of therapeutic intervention, can be extrapolated to human conditions [92]. Researchers can also explore innovative treatments, such as the promotion of revascularization and enhancement of limb salvage, in a preclinical setting using NO-boosted EVs. The use of female BALB/c mice provides standardization and uniformity, which are important for the consistent evaluation of therapeutic interventions and outcomes. The modification of MSC-derived EVs with NO nanocages enables nanovesicle regeneration for revascularization in CLI. This strategy can enhance the therapeutic potential of EVs by integrating NO nanocages to boost endothelial cell function and pericyte recruitment for vascular regeneration. This represents an effective and targeted approach for enhancing vascular regeneration and reducing amputations in patients with CLI [93]. The mouse model of hindlimb ischemia mimics the conditions of human CLI and can be used to assess the therapeutic effects of innovative treatments. Bioengineered stem cell membrane-coated nanocarriers (BSMNCs) loaded with VEGF have also been designed to leverage the angiogenic potential of VEGF and the regenerative properties of stem cells [94]. In prior research conducted to evaluate the therapeutic effects of artificial nanovesicles, systemic retro-orbital injections of BSMNCs loaded with VEGF were administered to mice with hindlimb ischemia [28]. Notable improvements were observed in key functions, such as muscle repair, limb salvage, and blood reperfusion, indicating that the BSMNCs were crucial for promoting the healing process. Laser Doppler imaging, a non-invasive method for the serial measurement of neovascularization and vascular outcomes in treated hindlimbs, was used for physiological assessments [95]. The BSMNC-treated mice exhibited significantly higher perfusion ratios, indicating successful neovascularization and improved blood flow in their ischemic limbs. This finding underscores the potential of BSMNCs as a promising therapeutic agent for CLI, highlighting their ability to enhance vascular repair and improve functional outcomes in ischemic conditions [96].

### 5.2. Functional and Molecular Outcomes in Preclinical Studies

In preclinical studies, MSC-EVs have shown significant potential for treating CLI by promoting angiogenesis and improving blood flow in ischemic limbs [97]. The functional outcomes of MSC-EVs include pro-regenerative effects, such as the enhancement of tissue regeneration and reduction in tissue damage, because of their ability to modulate inflammatory responses, enhance tissue repair processes, and promote cell survival, thereby improving outcomes in CLI models [98]. Proangiogenic abilities can be observed in EVs derived from serum (serum EVs). These EVs have been found to prevent muscle damage in acute hindlimb ischemia models and improve vascular remodeling [99]. sEVs (e-sEVs) have enhanced vascular remodeling, protected against muscle damage, and increased blood perfusion in ischemic hindlimbs, as evidenced by laser Doppler blood flow analysis and foot damage score evaluations [99,100]. Moreover, e-sEVs have significantly reduced the number of inflammatory cells in ischemic hindlimbs, as indicated by a reduction in inflammatory infiltrates compared to the control group in a prior study [101]. The molecular outcomes of MSC-derived EVs include the regulation of the expression of genes related to immune responses, tissue repair pathways, and inflammation, which can contribute to their therapeutic impact [99]. Artificial nanovesicles, such as mesosomes, have been engineered to mimic the functionality of MSCs. These nanovesicles aid in immunoregulation and tissue repair, which are important for regulating vascular regeneration [102]. In particular, mesosomes have significantly improved angiogenesis, mitigated inflammation, and reduced fibrosis in models of hindlimb ischemia [103]. Novel nanovesicles, such as a surface-coated copper-containing protein for NO release, have been developed for targeted delivery under ischemic conditions [104]. These nanovesicles have shown high revascularization effects in hindlimb ischemia models, underscoring their potential for enhancing vascular regeneration in CLI. Nanocarriers functionalized with engineered MSC membranes overexpressing CXCR4 have been found to enhance tropism toward ischemic tissues [105]. Upon integration with VEGF, these nanocarriers can significantly improve blood reperfusion, limb salvage, and tissue repair in CLI models. Targeted delivery of therapeutic cargo can ensure effective delivery of the cargo directly to the target areas [106].

### 5.3. Translation from Bench to Bedside

EVs serve as cell-free therapeutic agents. They provide a novel approach for treating CLI without the need for intact cells, potentially simplifying the therapeutic process [107]. Exosomes derived from various stem cells have exhibited significant potential for inducing angiogenesis in PAD models, unraveling a viable therapeutic option for addressing CLI. Preclinical studies have highlighted the angiogenic potential of exosomes, such as those derived from induced pluripotent stem cell (iPSC)-derived MSCs, which can enhance blood vessel formation in ischemic limbs [108,109]. The enrichment of EVs with angiogenic proteins (such as VEGF) and miRNAs (such as miRNA-210-3p) upregulates the expression of angiogenic genes in endothelial cells, thereby boosting tissue regeneration and blood vessel formation. Exosomes from specific stem cells, such as endometrium-derived MSCs, have exhibited efficacy in increasing microvessel density, indicating potential for significant vascular improvement in CLI. The translation of EV-based therapies from bench to bedside can provide opportunities for advancing treatment options for CLI [110]. This has been significantly observed in the treatment of CLI using BSMNCs. These nanovesicles are engineered to enhance ischemic tissue targeting and retention by inducing stem cell membranes, such as those derived from human adipose-derived stem cells [111,112,113]. This functionality significantly improves penetration across endothelial cell barriers and reduces uptake by immune cells, thereby potentially reducing the immune response and enhancing therapeutic efficacy. The customizability of these nanovesicles enables the creation of engineered biomimetic nanoparticles with hybrid functionalities. This translation from bench research to clinical application highlights the potential of artificial nanovesicles for revolutionizing CLI treatment and improving patient outcomes [100].

## 6. Clinical Trials and Emerging Therapies

### 6.1. Ongoing and Completed Clinical Trials

Several clinical trials have been conducted or are ongoing to evaluate the efficacy of EVs in CLI based on results from preclinical models. These trials consider key aspects, such as the characterization of the EV source, safety monitoring, and long-term adverse effects. Stem cell therapy, which mediates various processes within ischemic tissues, is crucial, emphasizing the importance of translational studies to evaluate emerging technologies for the development of cell therapies for CLI [114]. Ongoing clinical trials (e.g., NCT03968198, NCT04466007, and NCT04661644) and completed ones have investigated the efficacy of EVs in autologous peripheral blood mononuclear cell therapy for CLI and severe diabetic foot ulcers, aiming to restore limb perfusion and prevent major amputations in patients who are not eligible for revascularization procedures [115]. Key findings [Ethical Committee of IRCCS MultiMedica (protocol number 332.2018)] include a high frequency of sEVs in patients with a high level of TcPO_2_ after treatment, along with improvements in TcPO_2_ levels, ulcer healing, and walking capability after autologous peripheral blood mononuclear cell therapy. These findings collectively highlight the potential of EVs as a therapeutic target for improving tissue perfusion, promoting ulcer healing, and enhancing the quality of life of patients with CLI [116]. Ongoing clinical trials (e.g., NCT04173650 and completed ones (e.g., NCT02565264, NCT05191381, and NCT04493242) have also assessed the use of artificial nanovesicles for treating CLI. These trials aim to assess the efficacy and safety of nanovesicles in promoting tissue repair and angiogenesis in patients with CLI. Artificial nanovesicles designed to deliver specific therapeutic cargo, such as growth factors or therapeutic miRNAs, have resulted in enhanced angiogenesis and tissue regeneration in preclinical models [117]. Completed clinical trials (*Trial registration*: IRCT, IRCT20210221050446N1. Registered 9 May 2021) typically assess the safety, efficacy, and therapeutic potential of nanovesicles in promoting angiogenesis, muscle regeneration, and overall limb salvage in patients with CLI [118]. The key aspects evaluated include the optimal composition of proangiogenic and promyogenic factors in nanovesicles, their ability to recruit endothelial and skeletal muscle cells, and their efficacy in improving cell survival and function under ischemic and hyperglycemic conditions. The outcomes of these trials could significantly impact the development and clinical translation of nanovesicle-based therapies for CLI [50].

### 6.2. Challenges and Lessons Learned

Challenges in using EVs for treating CLI include standardizing the production techniques, ensuring consistent quality, and accurately quantifying and characterizing EVs. The critical concerns are understanding the pharmacokinetics of EVs, targeting them effectively to the ischemic limb, and ensuring their safety profiles [119]. Further clarification regarding optimal sources, administration routes, dosages, and treatment frequencies is required to achieve enhanced therapeutic effects [120]. Meta-analyses have indicated that EVs can improve outcomes in patients with diabetes-related CLI. MSC-EVs show potential by preventing muscle damage via neuregulin 1 (NRG1)-mediated signals, protecting muscle tissues and promoting angiogenesis. EVs can enhance myofiber regeneration, reduce inflammation in affected muscle tissues, and inhibit atrophy [121]. The main challenge in using artificial nanovesicles is the impact of inflammatory stimuli on their therapeutic efficacy. As CLI is characterized by severe inflammation, it can destabilize nanovesicles and alter their release profiles, thereby inhibiting their effectiveness [122]. Inflammatory stimuli, such as elevated levels of cytokines, ROS, and enzymes in inflamed tissues, can directly destabilize the lipid bilayer of nanovesicles, leading to premature drug release. Additionally, these stimuli can alter the pharmacokinetics and targeting efficiency of nanovesicles by increasing their uptake by immune cells or causing aggregation. Such disruptions not only reduce therapeutic efficacy, but also trigger off-target effects, emphasizing the need for designing nanovesicles with enhanced stability and inflammation-responsive properties [123]. Another challenge includes the selection of optimal therapeutic cargo, such as miRNAs, which are potent regulators of gene expression. The efficiency of miRNAs is context-dependent; they can effectively target specific molecular pathways associated with CLI. Standardizing the dosing and production of nanovesicles is also crucial [124]. The production of nanovesicles must be particularly standardized to ensure consistency in composition, size, and cargo loading. Variations in these parameters can lead to inconsistent therapeutic outcomes [125]. Determination of the appropriate dosing plan, including the dosage, frequency of administration, and delivery of nanovesicles, is challenging. Lessons learned from the use of artificial nanovesicles for CLI include the improvement in stability in inflammatory environments through various strategies, such as modifications of surface properties of nanovesicles, to resist degradation and enhance efficiency [126].

### 6.3. Future Directions for Clinical Applications

EVs are significant therapeutic armors that can treat CLI by transferring bioactive molecules and modulating cellular processes to enhance angiogenesis and tissue regeneration. Future studies should prioritize the identification of effective EVs, such MSC-EVs, to improve therapeutic outcomes and ensure patient safety [127]. Establishing standardized protocols for the isolation, characterization, and storage of EVs is essential to ensure consistency and reproducibility in their successful clinical applications. Long-term clinical trials are necessary to assess the safety and efficacy of EV therapy in CLI. These trials should focus on outcomes, such as limb preservation, wound healing, and pain management [128]. Such trials are crucial for monitoring the safety profile of EV therapy over extended periods, to ensure that no adverse effects or complications arise with prolonged treatment. They can evaluate limb preservation by tracking limb salvage rates, wound healing by measuring tissue regeneration, pain management by monitoring changes in pain levels, and quality of life by assessing physical function, emotional well-being, and overall satisfaction with treatment [129]. Long-term trials can provide insights into the sustained effects and durability of EV therapies by capturing trends, complications, and late-emerging outcomes [130]. Artificial nanovesicles show significant potential for treating CLI by enhancing therapeutic efficacy and biodistribution. Exosome-encapsulating gel constructs used for treating limb ischemia can be utilized to improve stability and retention in patients with CLI [131]. For example, the intramuscular injection of chitosan hydrogel-loaded exosomes into a limb ischemia model was found to enhance retention, reduce cell apoptosis, suppress fibrosis, and promote angiogenesis. Nanovesicles encapsulated in silk fibroin hydrogel exhibit sustained release, reducing vascular dysfunction in limb ischemia models. Addressing disturbances in calcium homeostasis in CLI is also crucial, as ischemia impairs ion pumps and reperfusion causes calcium influx, leading to cellular damage. Future studies should focus on optimizing nanovesicle stability, transport mechanisms, and biodistribution to enhance therapeutic outcomes in CLI [132,133,134].

## 7. Safety and Regulatory Considerations

### 7.1. Immunogenicity and Adverse Effects

Though the advancement and utilization of cell-derived EVs and artificial nanovesicles seem promising as treatment armors for CLI, the possible immunogenic effects need to be addressed. EVs are known to have low immunogenicity because of their endogenous origin. However, they may still elicit an immunogenic response in the host based on their source, the modifications performed, and the method of delivery [135]. These adverse effects may sometimes worsen the pre-existing condition via inflammation, unwanted immune activation, and systemic responses. To overcome these challenges, considerable in vitro and in vivo studies need to be conducted before proceeding to human trials. The treatment must align with the Food and Drug Administration (FDA) guidelines for immunogenicity testing [136].

### 7.2. Regulatory Landscape for EVs and Nanovesicles in CLI Therapy

The regulatory landscape around the therapeutic use of EVs and nanovesicles, specifically in the context of CLI, is continuously growing and dynamic [137]. Regulatory bodies, such as the U.S. FDA and the European Medicines Agency (EMA), play a crucial and defining role in ensuring the safety and efficacy of new medications. They have established rules and regulations for drug development agencies. These guidelines keep on changing because of the rapidly growing nature of the said field. EVs and nanovesicles undergo rigorous reviews before obtaining clinical approval, given their recent development as therapeutic agents [138]. The regulatory framework for these medicines often involves multiple phases, starting with preclinical assessment, during which their safety, effectiveness, and potential toxicities are thoroughly examined in animal models. Preclinical studies, such as those examining the therapeutic benefits of EVs derived from hypoxic HUVECs, have shown promise and safety in improving healing processes in CLI [139]. Such preclinical studies serve as a necessary step before proceeding to human trials. To obtain permission, regulatory agencies require the submission of extensive data on pharmacodynamics, pharmacokinetics, immunogenicity, and long-term adverse effects noted during clinical studies. Failure to do so usually leads to the rejection of the drug/treatment method [140]. Moreover, the regulatory rules require explicit documentation and uniformity in procedures, encompassing the techniques used for EV and nanovesicle separation, characterization, and purification. The repeatability of the said procedure is paramount without any inconsistency in the data. The data thus submitted are then extensively analyzed further. This is carried out to guarantee replication and maintain consistency across several batches. Ensuring the standardization of these components is crucial for minimizing any deviations that could potentially affect the safety and effectiveness of the therapy. Another major hurdle is the ever-evolving nature of the field of EVs, thus creating a knowledge gap between academia and industry worldwide. To adapt to the rapid technological advancements in EV and nanovesicle therapies for CLI, researchers, doctors, and regulatory authorities must engage in ongoing communication and modify regulatory frameworks that are stringent and effective [141].

## 8. Mechanistic Insights and Future Directions

### 8.1. Elucidating Mechanisms of EVs and Nanovesicles in CLI Therapy

**Figure 3:**
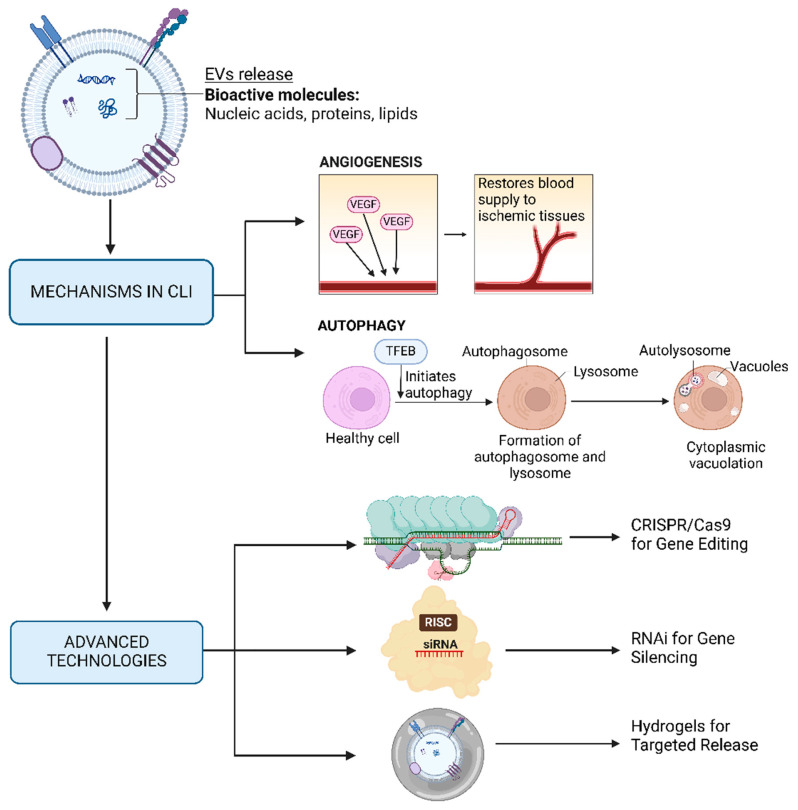
Mechanisms of extracellular vesicles in the treatment of CLI and implications for further advancements, Created with BioRender.com.

Cells release EVs and nanovesicles to regulate cell signaling and tissue restoration, particularly in conditions like CLI. These vesicles are employed to transport various bioactive molecules, such as proteins, lipids, and nucleic acids, in order to deliver messages to target cells, trigger specific cellular activities, and aid in repair mechanisms [142]. In the context of CLI, the therapeutic potential of EVs has been significantly enhanced by engineering them to express VEGF and TFEB [143]. The significance of EVs in CLI treatment is demonstrated in Figure 3, which extensively explains their importance. It provides a comprehensive overview of their therapeutic potential by highlighting their roles in facilitating tissue regeneration, regulating immunological responses, and transporting bioactive chemicals, and the implications for upcoming advancements in EV-based therapies. VEGF is crucial for angiogenesis, the process of forming new blood vessels, which is vital for restoring blood supply to ischemic tissues [144]. On the other hand, TFEB regulates autophagy, a process that clears damaged cells and promotes cell survival and tissue regeneration under ischemic conditions [28,145]. Nevertheless, considerable research is warranted to precisely determine the whole process of EV cargo sorting, release, and uptake [146]. This includes the identification of certain signaling molecules and the ways in which these signaling events occur. Knowledge in this direction will facilitate the development of more efficient therapies based on EVs, enabling the introduction of targeted therapies for ischemic tissues in CLI.

### 8.2. Harnessing Advanced Technologies for Therapeutic Optimization of EVs

With the help of novel biotechnological resources, the application of EVs and nanovesicles has dramatically increased in the field of therapy. This has been made possible by new techniques, such as CRISPR/Cas9 for gene editing, RNA interference (RNAi) for gene silencing, and advanced proteomics for detailed molecular protein analysis of EVs [147]. In other words, CRISPR/Cas9 can enable the modifications of genes in the cells that create EVs and guarantee that these vesicles contain proteins, RNA, or other molecules specific to particular diseases. Such precision engineering can enhance the specificity and efficacy of EV-based therapies. Hydrogels, as carriers of EVs, have been a major development for ensuring targeted and specific drug release. These hydrogels are responsive to stimuli. In other words, they will only release EVs at the right time based on environmental influences, such as pH level or temperature, to improve the therapeutic efficacy of the EVs [128]. From a methodological point of view, the incorporation of omics approaches, such as genomics, transcriptomics, proteomics, metabolomics, and lipidomics, into EV projects provides a broader perspective on how these structures function in paracrine signaling and pathophysiological processes. These numerous high-throughput omics technologies enable the precise identification of various components within EVs, including proteins, lipids, and RNA molecules, which bear evidence of the physiological and pathological climates of the cells from which they were secreted [148]. This multi-omics strategy can help choose potential biomarkers and drug targets, thereby improving the accuracy of EV-based medicine. For example, analyzing the proteomic and lipidomic profiles in CLI can help identify pathways and molecular cross-talks important for tissue repair and regeneration, which can be useful in designing targeted therapies. Such integrative analyses not only help to elucidate EV functions, but also open up opportunities for their use in personalized medicine when the targeted therapy is adjusted to the biological context of the patient [149,150]. Considering the current situation, wherein EV-based therapies for CLI are new and emerging, these latest technologies must be aligned to fit clinical practice. However, key challenges need to be addressed to act as a roadmap for establishing EV production strategies and protocols; these include several large-scale objectives, such as creating reliable processes for the large-scale production of EVs, developing a protocol on how to isolate and characterize EVs, and massive testing of therapies focusing on the safety and efficacy of EVs at both preclinical and clinical stages [151]. Significant efforts must also be directed toward other existing challenges, such as the stability of EVs, their delivery to the target, and the regulation of immune responses.

### 8.3. Personalized Approaches in Regenerative Medicine

Personalized regenerative therapy aims to customize treatments based on the characteristics of each patient, including their genetic makeup, disease status, and specific health conditions. This approach can significantly enhance the effectiveness of treatments by aligning them with each patient’s unique biological context [152]. Personalized therapeutic approaches for CLI could involve the use of patient-derived cells to produce autologous EVs. This strategy can minimize the risk of immune rejection and maximize the compatibility and effectiveness of the treatment. The characteristics of a patient’s genetic signature represent the molecular and cellular contexts within which therapies are delivered; therapies can therefore pinpoint and address the specific pathologies that underlie ischemia more accurately [153]. Nevertheless, to translate personalized regenerative medicine into clinical practice, there is a need to set up enhanced methods for the manufacture of personalized EVs and other regenerative treatments. This involves extending the current frontiers of stem cell research, improving bioengineering technology for patients, and developing better methods for personalized treatment planning [154]. Artificial nanovesicles and EVs are the most advanced therapeutic drugs that exhibit tremendous potential for treating complicated conditions, including CLI. Recent studies have revealed that both MSC-EVs and AD-MSC-EVs have proangiogenic activity, causing remarkable increases in tissue repair, vascular regeneration, and necrosis decrease. These vesicles carry bioactive molecules (such as VEGF) and specific microRNAs (such as miR-210-3p and miR-125b-5p). They can stimulate angiogenesis and endothelial cell proliferation by activating various pathways, such as the AKT/mTOR/HIF-1α and VEGF/VEGFR pathways. Improved delivery methods, such as the encapsulation of EVs in hydrogels, can enhance their stability and persistence at ischemic sites, thereby providing sustained therapeutic effects. In addition, through surface modification and adjunct therapies, bioengineered nanovesicles can improve targeting efficiency and treatment outcomes. Preclinical models have demonstrated the clinical potential of these vesicles for treating CLI by uniformly revealing improved limb function, vascular density, and blood flow. These findings are detailed in Table 2. Moreover, comprehensive genomic and proteomic profiling of patients with CLI can provide invaluable insights into the specific molecular targets for therapy, enabling clinicians to design more precise and effective treatment strategies. However, numerous barriers need to be overcome in order to translate these highly customized regenerative therapies from the laboratory to the clinic, including scalability issues, process standardization, approval from relevant authorities, and affordability for the patients. Overcoming these obstacles will necessitate an extensive collaboration between multidisciplinary teams, contemporary research activities, and the most crucial and challenging attempt to close the gap between innovation and practice. This concept shows great potential for the treatment of CLI because it involves targeting personalized solutions based on each patient’s specific characteristics. As evident from patient data, personalized therapeutic strategies are likely to yield better outcomes and may open the door to new trends in the niche of precision medicine [155].

## 9. Toward Clinical Translation and Practice

### 9.1. The Potential of EVs and Nanovesicles for CLI Revascularization

EVs and artificial nanovesicles have emerged as promising therapeutic modalities for the revascularization of CLI. Studies have revealed that EVs derived from stem cells, particularly MSC-EVs, possess potent proangiogenic properties. MSC-EVs may promote endothelial cell proliferation, migration, and tubular network formation, which are important stages in new vessel formation [156,157,161]. Moreover, EVs can specifically alter the immune response to reduce inflammation and increase tissue repair, thereby facilitating revascularization in patients with CLI [162]. Artificial nanovesicles can also be developed synthetically to mimic the characteristics of EVs. These vesicles can be engineered to hold a specific type of payload, such as growth factors, miRNAs, or small interfering RNAs, for delivery to the target tissue. Moreover, their surface characteristics can be altered to improve specific accumulation, systemic circulation time, and overall stability [163]. Such innovations in nanovesicle engineering have enormous potential for overcoming the constraints of conventional therapies and providing more effective and personalized treatment options for patients with CLI.

### 9.2. Challenges and Opportunities for Translation

Although EVs and nanovesicles have potential applications in many aspects of medicine, several challenges need to be addressed to improve their practical applications. A major challenge is the standardized production and characterization of EVs. Variability in EV isolation methods, sources, and characterization techniques can lead to inconsistencies in the quality and effectiveness of EV-based therapies [164]. Thus, the rigorous and careful selection of methods for EV isolation and characterization is indispensable, not only for generating interpretable preclinical or clinical data, but also for facilitating ongoing research. Another key challenge is related to the elucidation of the mode of action of EVs and nanovesicles. Despite the available data on their therapeutic effectiveness, their actual molecular mechanisms remain unclear [165]. Unraveling these mechanisms will help further develop and improve EV-based therapy and other vesicle-based treatments. Safety and immunogenicity are also significant concerns. Although EVs are considered biocompatible and non-immunogenic compared to synthetic nanoparticles, the risk of immune response persists, especially when they are derived from allogeneic sources [166]. Artificial nanovesicles can also pose new safety concerns because they are synthetic and may behave unpredictably in certain biological systems. Given the novel nature of these therapies, extensive preclinical studies must be coupled with strict surveillance in phase I and II trials to determine the safety standards of the therapies. Possible avenues for improving EV and nanovesicle therapies in CLI include the application of advances in bioengineering and nanotechnology. Some ideas include high-throughput methods for EV production, high-precision methods for cargo loading, and surface optimization of these vesicles to improve their function as therapeutic entities in targeting disease cells [167]. Moreover, the use of EVs and nanovesicles in combination with traditional therapeutic approaches, including pharmacological products or surgical procedures, may increase therapeutic efficacy.

### 9.3. Implications for Future Clinical Practice

This review indicates that the incorporation of EVs and artificial nanovesicles has renewed the possibility of improving the therapeutic options for CLI. It may potentially offer safer solutions to existing therapies, which require intricate operations and often cause severe side effects. By enhancing angiogenesis, modifying immune system reactions, and stimulating tissue regeneration, EVs and artificial (engineered) nanovesicles can increase limb preservation, alleviate pain, and ultimately improve the overall quality of life in patients with CLI [146,168]. Furthermore, the use of EV and nanovesicle therapies as individualized medicine supports the increasingly popular trend of precision or personalized medicine in modern medical practice. In this context, it is essential to discuss how the vesicle cargo and surface properties can be modulated to achieve the desired therapeutic effect, which may differ among patients. Compared to general approaches, such a specific treatment plan could potentially enhance therapeutic processes, increase effectiveness, and reduce possible adverse effects [169]. The successful translation of EV and nanovesicle therapies to clinical practice will require close collaboration among researchers, clinicians, and regulatory bodies. Establishing clear regulatory guidelines and ensuring thorough clinical evaluation are essential for the use of these innovative therapies for treating patients. Additionally, educating healthcare providers about the potential benefits and applications of EV- and nanovesicle-based treatments will be crucial for their adoption in clinical settings.

## 10. Conclusions

EVs and artificial nanovesicles represent a promising frontier for the treatment of CLI. Although challenges persist, ongoing research and technological advancements are paving the way for their clinical translation. These novel therapies have the potential to revolutionize the management of CLI, offering new hope for patients experiencing this debilitating condition.

## Figures and Tables

**Table 1:** List of classification systems for CLI depending on severity.

Classification	Description of Ischemia	Year	Ischemic Rest Pain	Ulcer	Gangrene
Fontaine [10]	Grading of ischemic symptoms.	1954	Yes, category 4/6	Class IV	Class IV
Rutherford [11]	AP < 40 mmHg and TP < 80 mmHg for rest pain. AP < 60 mmHg and TP < 40 mmHg for tissue loss.	1986	Yes, category III/IV	Category V	Category VI
Wagner [12]	Nil	1976	Nil	Grade 0: Pre- or post-ulcerative lesion	Grouping of ulcer and gangrene
UT [13]	ABI < 0.8 as the criterion for ischemia. No grade for ischemic severity.	1982	Nil	Yes, Grades 0–3	Nil
PEDIS [14]	Grade 1: No PAD symptoms, ABI > 0.9, and TBI > 0.6 mmHg. Grade 2: PAD symptoms, ABI < 0.9, AP > 50 mmHg, TP > 30 mmHg, and TcPO_2_ 30–60 mmHg. Grade 3: AP < 50 mmHg, TP < 30 mmHg, and TcPO_2_ < 30 mmHg.	2004	Nil	Yes, Grades 1–3	Nil

**Table 2:** Summary of key studies on the therapeutic potential of extracellular vesicles (EVs) and artificial nanovesicles in critical limb ischemia (CLI).

Serial No.	Title	Key Findings	References
1	Extracellular vesicles from mesenchymal stem cells activate VEGF receptors and accelerate recovery of hindlimb ischemia	MSC-EVs activate VEGF receptors, enhancing angiogenesis in ischemic limbs. MSC-EVs contain VEGF and miR-210-3p, which are crucial for angiogenesis. MSC-EVs increase blood reperfusion and new blood vessel formation in limbs. MSC-EVs stimulate downstream pathways, such as SRC, AKT, and ERK.	[144]
2	Exosomes from adipose-derived stem cells promote angiogenesis and reduce necrotic grade in hindlimb ischemia mouse models	ADSC-Exos reduce limb necrosis and stimulate angiogenesis in mice. ADSC-Exos enhance vascular regeneration and limb function recovery. ADSC-Exos improve SpO_2_ levels and muscle structure in treated mice. Exosomes accelerate blood circulation and reduce limb necrosis grade in mice.	[50]
3	Serum-derived extracellular vesicles (EVs) impact vascular remodeling and prevent muscle damage in acute hindlimb ischemia	sEVs improve vascular remodeling and prevent muscle damage in ischemia. e-sEVs enhance hindlimb perfusion and capillary density post surgery.	[99]
4	Lyophilized extracellular vesicles from adipose-derived stem cells increase muscle reperfusion but degrade muscle structural proteins in a mouse model of hindlimb ischemia–reperfusion injury	Lyophilized EVs enhance reperfusion but degrade muscle structural proteins. EVs increase the expression of the anti-inflammatory factor annexin A1 in skeletal muscle. EV treatment increases the levels of the inflammatory cytokines TNF-α and IL-6. The serum TNF-α level positively correlates with the reperfusion level after EV treatment. Alternative approaches, such as the targeting of mitochondrial permeability, may be more effective.	[31]
5	Extracellular vesicles from adipose stem cells prevent muscle damage and inflammation in a mouse model of hindlimb ischemia	ASC-EVs enhance muscle regeneration and protect muscle cells from damage. ASC-EVs promote myoblast proliferation and differentiation under ischemic conditions. NRG1 in ASC-EVs contributes to muscle protection and angiogenesis. ASC-EVs carry proangiogenic mRNAs and proteins for vascular growth. ASC-EVs exhibit anti-apoptotic effects in muscle cells.	[30]
6	Extracellular vesicles secreted by human urine-derived stem cells promote ischemia repair in a mouse model of hindlimb ischemia	USC-EVs significantly improve ischemic limb perfusion and function. USC-EVs promote angiogenesis and muscle regeneration. USC-EVs facilitate cell proliferation in vitro.	[24]
7	Enhanced therapeutic effects of mesenchymal stem cell-derived exosomes with an injectable hydrogel for hindlimb ischemia treatment	Chitosan hydrogel enhances exosome stability and retention, thereby exhibiting therapeutic effects. Exosomes in hydrogel show endothelium-protective and proangiogenic abilities in vitro. Hydrogel-incorporated exosomes improve therapeutic effects in hindlimb ischemia. Exosomes labeled with Gluc–lactadherin fusion proteins enhance retention post transplantation. Chitosan hydrogel acts as a barrier, protecting exosomes from immune clearance.	[27]
8	Multibiofunctional TFEB-engineered endothelial progenitor cell-derived extracellular vesicles/hydrogel system for rescuing critical limb ischemia	The TFEB–EVsFPD system rescues CLI by improving angiogenesis and muscle injury. FPD can be synthesized using PEI–F127–PEI and ODEX via a Schiff base reaction. The EVsFPD system responds to the severity of CLI through thermal and pH signals.	[143]
9	Exosomes derived from induced vascular progenitor cells promote angiogenesis in vitro and in an in vivo rat hindlimb ischemia model	iVPC-Exos promote angiogenesis in vitro, ex vivo, and in vivo. Exosomes contain proangiogenic proteins and microRNAs that induce vascular growth.	[46]
10	Exosomes derived from adipose-derived stem cells overexpressing glyoxalase-1 protect endothelial cells and enhance angiogenesis in type 2 diabetic mice with limb ischemia	G-ADSC-Exos enhance angiogenesis and protect endothelial cells. G-ADSC-Exos improve limb ischemia treatment efficiency in diabetic mice.	[112]
11	PDGF enhances the protective effect of adipose stem cell-derived extracellular vesicles in a model of acute hindlimb ischemia	PDGF-EVs enriched in anti-inflammatory factors protect muscle from acute ischemia. PDGF enhances ASC-EVs with TGF-b1 and IL-10 immunomodulatory proteins. PDGF-EVs reduce PBMC adhesion and promote Treg cell formation.	[113]
12	Enhanced pericyte–endothelial interactions through NO-boosted extracellular vesicles drive revascularization in a mouse model of ischemic injury	n-BANKs induce pericyte–endothelial interactions for complete revascularization in CLI. Enhanced NO production recruits pericytes, stabilizing vascular structures for revascularization.	[93]
13	Extracellular vesicles of ETV2-transfected fibroblasts stimulate endothelial cells and improve neovascularization in a murine model of hindlimb ischemia	EVs induce endothelial cell proliferation and enhance neovascularization in hindlimb ischemia. ETV2-transfected fibroblast-derived EVs show potential for blood vessel regeneration. EVs derived from ETV2-transduced fibroblasts improve neovascularization in murine models. Fibroblasts transduced with ETV-2 gene regulator stimulate endothelial cell proliferation.	[67]
14	Small extracellular vesicles of hypoxic endothelial cells regulate the therapeutic potential of adipose-derived mesenchymal stem cells via miR-486-5p/PTEN in a limb ischemia model	Hypoxic sEVs enhance ADSC resistance to ROS and improve angiogenic ability. Hypoxic sEVs downregulate PTEN via miR-486-5p, activating the AKT/MTOR/HIF-1α pathway. hsEV-primed ADSCs exhibit superior engraftment, angiogenesis, and tissue repair.	[54]
15	Hydrogel loaded with VEGF/TFEB-engineered extracellular vesicles for rescuing critical limb ischemia by a dual-pathway activation strategy	Engineered EV/hydrogel improves neovascularization and muscle recovery after CLI. Engineered EVs with hydrogel activate VEGF/VEGFR and autophagy–lysosomal pathways. Hydrogel enhances engineered EV stability and controls release at different temperatures.	[28]
16	Engineered extracellular vesicles with a high collagen-binding affinity present superior in situ retention and therapeutic efficacy in tissue repair	SILY-EVs enhance adhesion to collagen, improve retention, and promote tissue regeneration. SILY-EVs suppress inflammation and augment muscle regeneration in vivo. SILY-EVs exhibit the potential to enhance EV-mediated treatment efficacy in various diseases.	[55]
17	Delivery of miR-675 by stem cell-derived exosomes encapsulated in silk fibroin hydrogel prevents aging-induced vascular dysfunction in mouse hindlimb	miR-675 is downregulated in aging muscles and ischemic legs. miR-675 prevents aging by targeting the TGF–b1p21 pathway. Silk fibroin enhances the therapeutic effects of the miR-675 exosome. Exosomes encapsulated in silk fibroin promote blood perfusion.	[133]
18	Endothelial progenitor cell-derived microvesicles improve neovascularization in a murine model of hindlimb ischemia	EPC-derived MVs enhance neovascularization and recovery in a hindlimb ischemia model. MVs contain miR-126 and miR-296, promoting angiogenesis in mice. RNase-inactivated MVs reduce the proangiogenic effect of EPC-derived MVs. MVs improve limb perfusion and capillary density in the ischemic hindlimb. EPC-derived MVs can be used to treat peripheral arterial disease.	[156]
19	Angiogenic mechanisms of human CD34+ stem cell exosomes in the repair of ischemic hindlimb	CD34-Exos promote tissue repair by delivering angiomiR-126 to endothelial cells. CD34-Exos induce the proliferation of endothelial cells in post-ischemic hindlimb tissue. CD34-Exos have greater angiogenic and therapeutic efficacy than MNC-Exos.	[157]
20	Extracellular matrix hydrogel promotes tissue remodeling, arteriogenesis, and perfusion in a rat hindlimb ischemia model	ECM hydrogels increase tissue perfusion through arteriogenesis in rodent models. Skeletal muscle ECM hydrogel closely matches healthy tissue morphology. ECM hydrogel shifts inflammatory response and increases development, as evident in transcriptomic analysis.	[134]
21	Rejuvenation of senescent endothelial progenitor cells by EVs derived from mesenchymal stromal cells	EVs from young MSCs rejuvenate EPCs, mimicking MSC transplantation effects. Aged MSCs can be modified to produce EVs with enhanced rejuvenation. EVs offer a promising acellular therapeutic approach for cardiovascular diseases.	[58]
22	Adipose-derived stem cell-secreted exosomes enhance angiogenesis by promoting macrophage M2 polarization in type 2 diabetic mice with limb ischemia via the JAK/STAT6 pathway	ADSC-Exos induce M2 macrophage polarization via JAK/STAT6, enhancing angiogenesis. ADSC-Exos promote macrophage viability, migration, adhesion, and angiogenesis in diabetic mice. ADSC-Exos activate the JAK/STAT6 pathway, enhancing blood perfusion in diabetic limbs.	[51]
23	Co-delivery of bioengineered exosomes and oxygen for treating critical limb ischemia in diabetic mice	Exosomes and oxygen nanoparticles synergistically stimulate angiogenesis and muscle regeneration. Exosomes and oxygen nanoparticles improve cell survival, migration, and morphogenesis under hyperglycemic and ischemic conditions. Exosomes and oxygen nanoparticles promote angiogenesis and muscle regeneration in limbs.	[48]
24	Exosomal miR-125b-5p derived from adipose-derived mesenchymal stem cells enhances diabetic hindlimb ischemia repair via targeting alkaline ceramidase 2	ADSC-Exos enhance C2C12 cell proliferation and migration. ADSC-Exos promote angiogenesis in HUVECs. MiR-125b-5p from ADSC-Exos targets ACER2 for muscle repair. ADSC-Exos protect ischemic skeletal muscle and accelerate vascular regeneration.	[52]
25	Bradykinin-pretreated human cardiac-specific c-kit+ cells enhance exosomal miR-3059-5p and promote angiogenesis against hindlimb ischemia in mice	BK-c-kit+ exosomes enrich miR-3059-5p to promote angiogenesis in mice. miR-3059-5p suppresses TNFSF15, enhancing angiogenesis through the Akt/Erk1/2/Smad2/3 pathway. Exosomes from BK-c-kit cells promote tube formation in HUVECs. miR-3059-5p downregulates TNFSF15, facilitating angiogenesis in mice with hindlimb ischemia. BK-Exos upregulate miR-3059-5p, enhancing angiogenic function in c-kit cells.	[44]
26	Prophylactic exercise-derived circulating exosomal miR-125a-5p promotes endogenous revascularization after hindlimb ischemia by targeting endothelin-converting enzyme 1	Prophylactic exercise enhances revascularization after hindlimb ischemia through miR-125a-5p. Exercise promotes arteriogenesis and angiogenesis mediated by circulating exosomes.	[158]
27	Macrophage M2 polarization induced by exosomes from adipose-derived stem cells contributes to the exosomal proangiogenic effect on mouse ischemic hindlimb	Exosomes from ASCs induce M1 to M2 macrophage transition. M2 macrophages and ASC exosomes promote angiogenesis in ischemic hindlimbs.	[53]
28	Knockout of beta-2 microglobulin reduces stem cell-induced immune rejection and enhances ischemic hindlimb repair via exosome/miR-24/Bim pathway	B2M knockout enhances stem cell therapy for immune rejection. Exosome-based therapies show potential for treating tissue injury.	[90]
29	Exosomes secreted by human-induced pluripotent stem cell-derived mesenchymal stem cells attenuate limb ischemia by promoting angiogenesis in mice	iMSCs-Exos attenuate limb ischemia by promoting angiogenesis in mice. iMSCs-Exos enhance microvessel density and blood perfusion in ischemic limbs. iMSCs-Exos activate angiogenesis-related gene expression and protein secretion in HUVECs.	[159]
30	miR-709 exerts an angiogenic effect through an FGF2 upregulation induced by a GSK3B downregulation	miR-709 upregulates FGF2 by downregulating GSK3B in endothelial cells. miR-709 is a promising angiogenic microRNA in hindlimb ischemia. Aortic ring assay revealed 14 miRNAs with angiogenic potential. EVs containing miR-709 can increase FGF2 mRNA expression in thigh tissues.	[45]
31	Extracellular vesicles derived from hypoxia-preconditioned bone marrow mesenchymal stem cells ameliorate lower limb ischemia by delivering miR-34c	Hyp-EVs improve blood flow and capillary density in lower limb ischemia. miR-34c promotes M2 macrophage polarization and anti-inflammatory cytokine production. PTEN silencing facilitates M2 macrophage polarization in lower limb ischemia.	[39]
32	Tracking radiolabeled endothelial microvesicles predicts their therapeutic efficacy: a proof-of-concept study in peripheral ischemia mouse models using SPECT/CT imaging	LEVs are homed to ischemic limbs and correlate with reperfusion intensity, improving motility. Radiolabeled LEVs were tracked in vivo and quantified to assess whole-body distribution. LEVs show early and specific homing to ischemic hindlimbs.	[160]
33	Platelet extracellular vesicles enhance the proangiogenic potential of adipose-derived stem cells in vivo and in vitro	Platelet EVs enhance the proangiogenic potential of adipose-derived stem cells. Microparticles act as “cargo”, facilitating the migration of implanted cells.	[31]
34	Stem cell-derived exosomes prevent pyroptosis and repair ischemic muscle injury through a novel exosome/circHIPK3/FOXO3a pathway	UMSC-Exo treatment improves muscle function by releasing circHIPK3. UMSC-Exos inhibit pyroptosis and inflammasome activation in ischemic muscle. circHIPK3 downregulates miR-421, thereby increasing FOXO3a expression. Exosomes prevent pyroptosis and enhance muscle recovery in ischemic injury.	[25]
35	XBP1 splicing triggers miR-150 transfer from smooth muscle cells to endothelial cells via extracellular vesicles	XBP1 deficiency in SMCs reduces angiogenesis in ischemic tissues. SMC-derived EVs control EC migration through miR-150 transfer. PDGF-induced SMCs secrete miR-150-containing EVs via XBP1 splicing.	[28]

## Data Availability

No new data were created or analyzed in this study.

## References

[1] Uccioli L., Meloni M., Izzo V., Giurato L., Merolla S., Gandini R. (2018). Critical limb ischemia: Current challenges and future prospects. Vasc. Health Risk Manag..

[2] Fabiani I., Calogero E., Pugliese N.R., Di Stefano R., Nicastro I., Buttitta F., Nuti M., Violo C., Giannini D., Morgantini A. (2018). Critical Limb Ischemia: A Practical Up-To-Date Review. Angiology.

[3] Schanzer A., Conte M.S. (2010). Critical Limb Ischemia. Curr. Treat. Options Cardiovasc. Med..

[4] Sharma A. (2019). Current review with evolving management strategies in critical limb ischemia. Indian J. Radiol. Imaging.

[5] Farber A., Eberhardt R.T. (2016). The Current State of Critical Limb Ischemia: A Systematic Review. JAMA Surg..

[6] Duff S., Mafilios M.S., Bhounsule P., Hasegawa J.T. (2019). The burden of critical limb ischemia: A review of recent literature. Vasc. Health Risk Manag..

[7] Kinlay S. (2016). Management of Critical Limb Ischemia. Circ. Cardiovasc. Interv..

[8] Bolton L. (2019). Peripheral arterial disease: Scoping review of patient-centred outcomes. Int. Wound J..

[9] Cao P., Eckstein H., De Rango P., Setacci C., Ricco J.-B., de Donato G., Becker F., Robert-Ebadi H., Diehm N., Schmidli J. (2011). Chapter II: Diagnostic methods. Eur. J. Vasc. Endovasc. Surg..

[10] Fontaine R., Kim M., Kieny R. (1954). Surgical treatment of peripheral circulation disorders. Helv. Chir. Acta.

[11] Rutherford R.B., Baker J., Ernst C., Johnston K., Porter J.M., Ahn S., Jones D.N. (1997). Recommended standards for reports dealing with lower extremity ischemia: Revised version. J. Vasc. Surg..

[12] Wagner F.W. (1981). The dysvascular foot: A system for diagnosis and treatment. Foot Ankle.

[13] Armstrong D.G., Lavery L.A., Harkless L.B. (1998). Validation of a diabetic wound classification system. The contribution of depth, infection, and ischemia to risk of amputation. Diabetes Care.

[14] Schaper N.C. (2004). Diabetic foot ulcer classification system for research purposes: A progress report on criteria for including patients in research studies. Diabetes Metab. Res. Rev..

[15] Adam D.J., Beard J.D., Cleveland T., Bell J., Bradbury A.W., Forbes J.F., Fowkes F.G.R., Gillepsie I., Ruckley C.V., Raab G. (2005). Bypass versus angioplasty in severe ischaemia of the leg (BASIL): Multicentre, randomised controlled trial. Lancet.

[16] Yannoutsos A., Gaïsset R., Lazareth I. (2022). Challenges in the management of patients with critical limb ischemia. J. Med. Vasc..

[17] Cambou J., Aboyans V., Constans J., Lacroix P., Dentans C., Bura A. (2010). Characteristics and Outcome of Patients Hospitalised for Lower Extremity Peripheral Artery Disease in France: The COPART Registry. Eur. J. Vasc. Endovasc. Surg..

[18] Di Primio M., Angelopoulos G., Lazareth I., Lin F., Petit A., Priollet P., Sapoval M., Emmerich J., Yannoutsos A. (2019). Endovascular Extra-Anatomic Femoro-Popliteal Bypass for Limb Salvage in Chronic Critical Limb Ischemia. Cardiovasc. Interv. Radiol..

[19] Yannoutsos A., Lin F., Billuart O., Buronfosse A., Sacco E., Beaussier H., Mourad J.-J., Emmerich J., Lazareth I., Priollet P. (2021). Low admission blood pressure as a marker of poor 1-year survival in patients with revascularized critical limb ischemia. J. Hypertens..

[20] Agarwal S., Pitcavage J.M., Sud K., Thakkar B. (2017). Burden of Readmissions Among Patients With Critical Limb Ischemia. J. Am. Coll. Cardiol..

[21] Paul M.K. (2022). Extracellular Vesicles [Working Title].

[22] Nguyen J., Fuhrmann G. (2022). Extracellular Vesicles—A Versatile Biomaterial. Adv. Healthc. Mater..

[23] Zhang K., Li Z. (2019). Molecular Imaging of Therapeutic Effect of Mesenchymal Stem Cell-Derived Exosomes for Hindlimb Ischemia Treatment. Methods Mol. Biol..

[24] Zhu Q., Li Q., Niu X., Zhang G., Ling X., Zhang J., Wang Y., Deng Z. (2018). Extracellular Vesicles Secreted by Human Urine-Derived Stem Cells Promote Ischemia Repair in a Mouse Model of Hind-Limb Ischemia. Cell. Physiol. Biochem..

[25] Yan B., Zhang Y., Liang C., Liu B., Ding F., Wang Y., Zhu B., Zhao R., Yu X.-Y., Li Y. (2020). Stem cell-derived exosomes prevent pyroptosis and repair ischemic muscle injury through a novel exosome/circHIPK3/FOXO3a pathway. Theranostics.

[26] Xing Z., Zhao C., Wu S., Yang D., Zhang C., Wei X., Wei X., Su H., Liu H., Fan Y. (2022). Hydrogel Loaded with VEGF/TFEB-Engineered Extracellular Vesicles for Rescuing Critical Limb Ischemia by a Dual-Pathway Activation Strategy. Adv. Healthc. Mater..

[27] Zhang K., Zhao X., Chen X., Wei Y., Du W., Wang Y., Liu L., Zhao W., Han Z., Kong D. (2018). Enhanced Therapeutic Effects of Mesenchymal Stem Cell-Derived Exosomes with an Injectable Hydrogel for Hindlimb Ischemia Treatment. ACS Appl. Mater. Interfaces.

[28] Zhao Y., Li Y., Luo P., Gao Y., Yang J., Lao K.-H., Wang G., Cockerill G., Hu Y., Xu Q. (2016). XBP1 splicing triggers miR-150 transfer from smooth muscle cells to endothelial cells via extracellular vesicles. Sci. Rep..

[29] Mendhe B., Khan M.B., Dunwody D., El Baradie K.B.Y., Smith K., Zhi W., Sharma A., Lee T.J., Hamrick M.W. (2023). Lyophilized Extracellular Vesicles from Adipose-Derived Stem Cells Increase Muscle Reperfusion but Degrade Muscle Structural Proteins in a Mouse Model of Hindlimb Ischemia-Reperfusion Injury. Cells.

[30] Federico F., Andrea R., Cristina G., Massimo C., Marta T., Claudia C., Andrea R., Gabriele T., Saveria F., Vittoria G.M. (2020). Extracellular Vesicles From Adipose Stem Cells Prevent Muscle Damage and Inflammation in a Mouse Model of Hind Limb Ischemia: Role of Neuregulin-1. Arterioscler. Thromb. Vasc. Biol..

[31] Tang Y., Li J., Wang W., Chen B., Chen J., Shen Z., Hou J., Mei Y., Liu S., Zhang L. (2021). Platelet extracellular vesicles enhance the proangiogenic potential of adipose-derived stem cells in vivo and in vitro. Stem Cell Res. Ther..

[32] Jia Y., Yu L., Ma T., Xu W., Qian H., Sun Y., Shi H. (2022). Small extracellular vesicles isolation and separation: Current techniques, pending questions and clinical applications. Theranostics.

[33] Pols M.S., Klumperman J. (2009). Trafficking and function of the tetraspanin CD63. Exp. Cell Res..

[34] Wollert T., Hurley J.H. (2010). Molecular mechanism of multivesicular body biogenesis by ESCRT complexes. Nature.

[35] Akers J.C., Gonda D., Kim R., Carter B.S., Chen C.C. (2013). Biogenesis of extracellular vesicles (EV): Exosomes, microvesicles, retrovirus-like vesicles, and apoptotic bodies. J. Neuro-Oncol..

[36] Abels E.R., Breakefield X.O. (2016). Introduction to Extracellular Vesicles: Biogenesis, RNA Cargo Selection, Content, Release, and Uptake. Cell. Mol. Neurobiol..

[37] Bacakova L., Zarubova J., Travnickova M., Musilkova J., Pajorova J., Slepicka P., Kasalkova N.S., Svorcik V., Kolska Z., Motarjemi H. (2018). Stem cells: Their source, potency and use in regenerative therapies with focus on adipose-derived stem cells—A review. Biotechnol. Adv..

[38] Desrochers L.M., Bordeleau F., Reinhart-King C.A., Cerione R.A., Antonyak M.A. (2016). Microvesicles provide a mechanism for intercellular communication by embryonic stem cells during embryo implantation. Nat. Commun..

[39] Peng X., Liu J., Ren L., Liang B., Wang H., Hou J., Yuan Q. (2023). Extracellular vesicles derived from hypoxia-preconditioned bone marrow mesenchymal stem cells ameliorate lower limb ischemia by delivering miR-34c. Mol. Cell. Biochem..

[40] Wu R., Gao W., Yao K., Ge J. (2019). Roles of exosomes derived from immune cells in cardiovascular diseases. Front. Immunol..

[41] de la Torre Gomez C., Goreham R.V., Bech Serra J.J., Nann T., Kussmann M. (2018). “Exosomics”-A review of biophysics, biology and biochemistry of exosomes with a focus on human breast milk. Front. Genet..

[42] Chopra N., Arya B.D., Jain N., Yadav P., Wajid S., Singh S.P., Choudhury S. (2019). Biophysical Characterization and Drug Delivery Potential of Exosomes from Human Wharton’s Jelly-Derived Mesenchymal Stem Cells. ACS Omega.

[43] Kesidou D., da Costa Martins P.A., De Windt L.J., Brittan M., Beqqali A., Baker A.H. (2020). Extracellular Vesicle miRNAs in the Promotion of Cardiac Neovascularisation. Front. Physiol..

[44] Li J., Song F., Chen R., Yang J., Liu J., Huang L., Duan F., Kou M., Lian B.X., Zhou X. (2023). Bradykinin-pretreated Human cardiac-specific c-kit+ Cells Enhance Exosomal miR-3059-5p and Promote Angiogenesis Against Hindlimb Ischemia in mice. Stem Cell Rev. Rep..

[45] Ueno K., Kurazumi H., Suzuki R., Yanagihara M., Mizoguchi T., Harada T., Morikage N., Hamano K. (2024). miR-709 exerts an angiogenic effect through a FGF2 upregulation induced by a GSK3B downregulation. Sci. Rep..

[46] Johnson T.K., Zhao L., Zhu D., Wang Y., Xiao Y., Oguljahan B., Zhao X., Kirlin W.G., Yin L., Chilian W.M. (2019). Exosomes derived from induced vascular progenitor cells promote angiogenesis in vitro and in an in vivo rat hindlimb ischemia model. Am. J. Physiol. Heart Circ. Physiol..

[47] Berger M.M., Macholz F., Mairbäurl H., Bärtsch P. (2015). Remote ischemic preconditioning for prevention of high-altitude diseases: Fact or fiction?. J. Appl. Physiol..

[48] Zhong T., Gao N., Guan Y., Liu Z., Guan J. (2023). Co-Delivery of Bioengineered Exosomes and Oxygen for Treating Critical Limb Ischemia in Diabetic Mice. ACS Nano.

[49] Vicencio J.M., Yellon D.M., Sivaraman V., Das D., Boi-Doku C., Arjun S., Zheng Y., Riquelme J.A., Kearney J., Sharma V. (2015). Plasma exosomes protect the myocardium from ischemia-reperfusion injury. J. Am. Coll. Cardiol..

[50] Nguyen T.H.-N., Van Pham P., Vu N.B. (2023). Exosomes from adipose-derived stem cells promote angiogenesis and reduce necrotic grade in hindlimb ischemia mouse models. Iran. J. Basic Med. Sci..

[51] Wang X., Chen S., Lu R., Sun Y., Song T., Nie Z., Yu C., Gao Y. (2022). Adipose-derived stem cell-secreted exosomes enhance angiogenesis by promoting macrophage M2 polarization in type 2 diabetic mice with limb ischemia via the JAK/STAT6 pathway. Heliyon.

[52] Guo J., Yang X., Chen J., Wang C., Sun Y., Yan C., Ren S., Xiong H., Xiang K., Zhang M. (2023). Exosomal miR-125b-5p derived from adipose-derived mesenchymal stem cells enhance diabetic hindlimb ischemia repair via targeting alkaline ceramidase 2. J. Nanobiotechnol..

[53] Zhu D., Johnson T.K., Wang Y., Thomas M., Huynh K., Yang Q., Bond V.C., Chen Y.E., Liu D. (2020). Macrophage M2 polarization induced by exosomes from adipose-derived stem cells contributes to the exosomal proangiogenic effect on mouse ischemic hindlimb. Stem Cell Res. Ther..

[54] Shen Z., Wang W., Chen J., Chen B., Tang Y., Hou J., Li J., Liu S., Mei Y., Zhang L. (2022). Small extracellular vesicles of hypoxic endothelial cells regulate the therapeutic potential of adipose-derived mesenchymal stem cells via miR-486-5p/PTEN in a limb ischemia model. J. Nanobiotechnol..

[55] Hao D., Lu L., Song H., Duan Y., Chen J., Carney R., Li J.J., Zhou P., Nolta J., Lam K.S. (2022). Engineered extracellular vesicles with high collagen-binding affinity present superior in situ retention and therapeutic efficacy in tissue repair. Theranostics.

[56] Qu Q., Fu B., Long Y., Liu Z.-Y., Tian X.-H. (2023). Current Strategies for Promoting the Large-scale Production of Exosomes. Curr. Neuropharmacol..

[57] Du W., Zhang K., Zhang S., Wang R., Nie Y., Tao H., Han Z., Liang L., Wang D., Liu J. (2017). Enhanced proangiogenic potential of mesenchymal stem cell-derived exosomes stimulated by a nitric oxide releasing polymer. Biomaterials.

[58] Wang L., Wei J., Ferreira A.D.F., Wang H., Zhang L., Zhang Q., Bellio M.A., Chu X.-M., Khan A., Jayaweera D. (2020). Rejuvenation of Senescent Endothelial Progenitor Cells by Extracellular Vesicles Derived From Mesenchymal Stromal Cells. JACC Basic Transl. Sci..

[59] Shi S., Li T., Wen X., Wu S.Y., Xiong C., Zhao J., Lincha V.R., Chow D.S., Liu Y., Sood A.K. (2019). Copper-64 Labeled PEGylated Exosomes for In Vivo Positron Emission Tomography and Enhanced Tumor Retention. Bioconjugate Chem..

[60] Islam K., Razizadeh M., Liu Y. (2023). Coarse-Grained Molecular Simulation of Extracellular Vesicles Squeezing for Drug Loading. Phys. Chem. Chem. Phys..

[61] Zeng H., Guo S., Ren X., Wu Z., Liu S., Yao X. (2023). Current Strategies for Exosome Cargo Loading and Targeting Delivery. Cells.

[62] Zhu S., Huang H., Liu D., Wen S., Shen L., Lin Q. (2022). Augmented cellular uptake and homologous targeting of exosome-based drug loaded IOL for posterior capsular opacification prevention and biosafety improvement. Bioact. Mater..

[63] Yerneni S.S., Yalcintas E.P., Smith J.D., Averick S., Campbell P.G., Ozdoganlar O.B. (2022). Skin-targeted delivery of extracellular vesicle-encapsulated curcumin using dissolvable microneedle arrays. Acta Biomater..

[64] Wang C., Li N., Li Y., Hou S., Zhang W., Meng Z., Wang S., Jia Q., Tan J., Wang R. (2022). Engineering a HEK-293T exosome-based delivery platform for efficient tumor-targeting chemotherapy/internal irradiation combination therapy. J. Nanobiotechnol..

[65] Liang Y., Duan L., Lu J., Xia J. (2021). Engineering exosomes for targeted drug delivery. Theranostics.

[66] Ansari A., Hussain A., Wadekar R., Malik A., Mujtaba A., Ansari M.Y., Siddique M.U.M., Goyal S.N. (2023). Nanovesicles based drug targeting to control tumor growth and metastasis. Adv. Cancer Biol.-Metastasis.

[67] Van Pham P., Vu N.B., Dao T.T.-T., Le H.T.-N., Phi L.T., Huynh O.T., Truong M.T.-H., Nguyen O.T.-K., Phan N.K. (2017). Extracellular vesicles of ETV2 transfected fibroblasts stimulate endothelial cells and improve neovascularization in a murine model of hindlimb ischemia. Cytotechnology.

[68] Lee J., Sim W., Park H., Park B., Joung Y.K. (2023). Targeted Delivery of Apoptotic Cell-Derived Nanovesicles prevents Cardiac Remodeling and Attenuates Cardiac Function Exacerbation. Adv. Funct. Mater..

[69] Karpuz M., İlhan M., Gültekin H.E., Ozgenc E., Şenyiğit Z., Atlihan-Gundogdu E. (2022). Nanovesicles for tumor-targeted drug delivery. Appl. Nanovesicular Drug Deliv..

[70] Gandek T.B., van der Koog L., Nagelkerke A. (2023). A Comparison of Cellular Uptake Mechanisms, Delivery Efficacy, and Intracellular Fate between Liposomes and Extracellular Vesicles. Adv. Healthc. Mater..

[71] Ren Y., Nie L., Zhu S., Zhang X. (2022). Nanovesicles-Mediated Drug Delivery for Oral Bioavailability Enhancement. Int. J. Nanomed..

[72] Ou Y.-H., Liang J., Chng W.H., Muthuramalingam R.P.K., Ng Z.X., Lee C.K., Neupane Y.R., Yau J.N.N., Zhang S., Lou C.K.L. (2022). Investigations on Cellular Uptake Mechanisms and Immunogenicity Profile of Novel Bio-Hybrid Nanovesicles. Pharmaceutics.

[73] Elena M., Eleftheria G., Yiannis S., Lefteris Z.C., Michael P., Georgios A., Christos P.C. (2022). Clinical trials of nanovesicles for drug delivery applications. Appl. Nanovesicular Drug Deliv..

[74] Massaro C., Sgueglia G., Frattolillo V., Baglio S.R., Altucci L., Dell’Aversana C. (2020). Extracellular vesicle-based nucleic acid delivery: Current advances and future perspectives in cancer therapeutic strategies. Pharmaceutics.

[75] Record M., Carayon K., Poirot M., Silvente-Poirot S. (2014). Exosomes as new vesicular lipid transporters involved in cell-cell communication and various pathophysiologies. Biochim. Biophys. Acta.

[76] Palanisamy C.P., Pei J.J., Alugoju P., Anthikapalli N.V.A., Jayaraman S., Veeraraghavan V.P., Gopathy S., Roy J.R., Janaki C.S., Thalamati D. (2023). New strategies of neurodegenerative disease treatment with extracellular vesicles (EVs) derived from mesenchymal stem cells (MSCs). Theranostics.

[77] Papareddy P., Tapken I., Kroh K., Bhongir R.K.V., Rahman M., Baumgarten M., Cim E.I., Györffy L., Smeds E., Neumann A. (2024). The role of extracellular vesicle fusion with target cells in triggering systemic inflammation. Nat. Commun..

[78] ZKwok Z.H., Wang C., Jin Y. (2021). Extracellular Vesicle Transportation and Uptake by Recipient Cells: A Critical Process to Regulate Human Diseases. Processes.

[79] Salomon C., Das S., Erdbrügger U., Kalluri R., Lim S.K., Olefsky J.M., Rice G.E., Sahoo S., Tao W.A., Vader P. (2022). Extracellular Vesicles and Their Emerging Roles as Cellular Messengers in Endocrinology: An Endocrine Society Scientific Statement. Endocr. Rev..

[80] Yáñez-Mó M., Siljander P.R.-M., Andreu Z., Bedina Zavec A., Borràs F.E., Buzas E.I., Buzas K., Casal E., Cappello F., Carvalho J. (2015). Biological properties of extracellular vesicles and their physiological functions. J. Extracell. Vesicles.

[81] Cheng W., Xu C., Su Y., Shen Y., Yang Q., Zhao Y., Zhao Y., Liu Y. (2023). Engineered Extracellular Vesicles: A potential treatment for regeneration. iScience.

[82] Çelik P.A., Erdogan-Gover K., Barut D., Enuh B.M., Amasya G., Sengel-Türk C.T., Derkus B., Çabuk A. (2023). Bacterial Membrane Vesicles as Smart Drug Delivery and Carrier Systems: A New Nanosystems Tool for Current Anticancer and Antimicrobial Therapy. Pharmaceutics.

[83] Fan Z., Jiang C., Wang Y., Wang K., Marsh J., Zhang D., Chen X., Nie L. (2022). Engineered extracellular vesicles as intelligent nanosystems for next-generation nanomedicine. Nanoscale Horiz..

[84] Al-Jipouri A., Almurisi S.H., Al-Japairai K., Bakar L.M., Doolaanea A.A. (2023). Liposomes or Extracellular Vesicles: A Comprehensive Comparison of Both Lipid Bilayer Vesicles for Pulmonary Drug Delivery. Polymers.

[85] Dang X.T.T., Kavishka J.M., Zhang D.X., Pirisinu M., Le M.T.N. (2020). Extracellular Vesicles as an Efficient and Versatile System for Drug Delivery. Cells.

[86] Zhang B., Tian X., Hao J., Xu G., Zhang W. (2020). Mesenchymal Stem Cell-Derived Extracellular Vesicles in Tissue Regeneration. Cell Transplant..

[87] Herrmann I.K., Wood M.J.A., Fuhrmann G. (2021). Extracellular vesicles as a next-generation drug delivery platform. Nat. Nanotechnol..

[88] Chavda V.P., Pandya A., Kumar L., Raval N., Vora L.K., Pulakkat S., Patravale V., Duo Y., Tang B.Z. (2023). Exosome nanovesicles: A potential carrier for therapeutic delivery. Nano Today.

[89] Aref Z., De Vries M.R., Quax P.H.A. (2019). Variations in Surgical Procedures for Inducing Hind Limb Ischemia in Mice and the Impact of These Variations on Neovascularization Assessment. Int. J. Mol. Sci..

[90] Zhang Y., Wang Y., Shao L., Pan X., Liang C., Liu B., Zhang Y., Xie W., Yan B., Liu F. (2020). Knockout of beta-2 microglobulin reduces stem cell-induced immune rejection and enhances ischaemic hindlimb repair via exosome/miR-24/Bim pathway. J. Cell. Mol. Med..

[91] Misra S., Shishehbor M.H., Takahashi E.A., Aronow H.D., Brewster L.P., Bunte M.C., Kim E.S., Lindner J.R., Rich K., On behalf of the American Heart Association Council on Peripheral Vascular Disease (2019). Perfusion Assessment in Critical Limb Ischemia: Principles for Understanding and the Development of Evidence and Evaluation of Devices: A Scientific Statement From the American Heart Association. Circulation.

[92] Perlman R.L. (2016). Mouse models of human disease: An evolutionary perspective. Evol. Med. Public Health.

[93] Guo L., Yang Q., Wei R., Zhang W., Yin N., Chen Y., Xu C., Li C., Carney R.P., Li Y. (2023). Enhanced pericyte-endothelial interactions through NO-boosted extracellular vesicles drive revascularization in a mouse model of ischemic injury. Nat. Commun..

[94] Bose R.J., Ha K., McCarthy J.R. (2021). Bio-inspired nanomaterials as novel options for the treatment of cardiovascular disease. Drug Discov. Today.

[95] Cowled P., Fitridge R. (2011). Pathophysiology of Reperfusion Injury. Mechanisms of Vascular Disease a Reference Book for Vascular Specialists.

[96] Bose R.J., Kim B.J., Arai Y., Han I.-B., Moon J.J., Paulmurugan R., Park H., Lee S.-H. (2018). Bioengineered stem cell membrane functionalized nanocarriers for therapeutic targeting of severe hindlimb ischemia. Biomaterials.

[97] Elshaer S.L., Bahram S.H., Rajashekar P., Gangaraju R., El-Remessy A.B. (2022). Modulation of Mesenchymal Stem Cells for Enhanced Therapeutic Utility in Ischemic Vascular Diseases. Int. J. Mol. Sci..

[98] Ciferri M.C., Quarto R., Tasso R. (2021). Extracellular Vesicles as Biomarkers and Therapeutic Tools: From Pre-Clinical to Clinical Applications. Biology.

[99] Cavallari C., Ranghino A., Tapparo M., Cedrino M., Figliolini F., Grange C., Giannachi V., Garneri P., Deregibus M.C., Collino F. (2017). Serum-derived extracellular vesicles (EVs) impact on vascular remodeling and prevent muscle damage in acute hind limb ischemia. Sci. Rep..

[100] Łabędź-Masłowska A., Vergori L., Kędracka-Krok S., Karnas E., Bobis-Wozowicz S., Sekuła-Stryjewska M., Sarna M., Andriantsitohaina R., Zuba-Surma E.K. (2024). Mesenchymal stem cell-derived extracellular vesicles exert pro-angiogenic and pro-lymphangiogenic effects in ischemic tissues by transferring various microRNAs and proteins including ITGa5 and NRP1. J. Nanobiotechnol..

[101] Richards J., Gabunia K., Kelemen S.E., Kako F., Choi E.T., Autieri M.V. (2015). Interleukin-19 increases Angiogenesis in ischemic hind limbs by Direct Effects on both Endothelial Cells and Macrophage Polarization. J. Mol. Cell. Cardiol..

[102] Yin T., Liu Y., Ji W., Zhuang J., Chen X., Gong B., Chu J., Liang W., Gao J., Yin Y. (2023). Engineered mesenchymal stem cell-derived extracellular vesicles: A state-of-the-art multifunctional weapon against Alzheimer’s disease. Theranostics.

[103] Cooke J.P., Losordo D.W. (2015). Modulating the Vascular Response to Limb Ischemia Angiogenic and Cell Therapies. Circ. Res..

[104] Picone P., Palumbo F.S., Federico S., Pitarresi G., Adamo G., Bongiovanni A., Chaves A., Cancemi P., Muccilli V., Giglio V. (2021). Nano-structured myelin: New nanovesicles for targeted delivery to white matter and microglia, from brain-to-brain. Mater. Today Bio.

[105] Shirbaghaee Z., Hassani M., Keshel S.H., Soleimani M. (2022). Emerging roles of mesenchymal stem cell therapy in patients with critical limb ischemia. Stem Cell Res. Ther..

[106] Zhuang J., Zhang X., Liu Q., Zhu M., Huang X. (2022). Targeted delivery of nanomedicines for promoting vascular regeneration in ischemic diseases. Theranostics.

[107] Galieva L.R., James V., Mukhamedshina Y.O., Rizvanov A.A. (2019). Therapeutic Potential of Extracellular Vesicles for the Treatment of Nerve Disorders. Front. Neurosci..

[108] Xiao Y., Zhang Y., Li Y., Peng N., Liu Q., Qiu D., Cho J., Borlongan C.V., Yu G. (2022). Exosomes Derived From Mesenchymal Stem Cells Pretreated With Ischemic Rat Heart Extracts Promote Angiogenesis via the Delivery of DMBT1. Cell Transplant..

[109] Huang Z., Chen Z., Ye T., Luo L., Zhang J., Li Q., Wang Y., Zhao B. (2024). Large extracellular vesicles from induced pluripotent stem cell-marrow stem cells enhance limb angiogenesis via ERK/MAPK. Nanomedicine.

[110] Babaei M., Rezaie J. (2021). Application of stem cell-derived exosomes in ischemic diseases: Opportunity and limitations. J. Transl. Med..

[111] Jeyaraman M., Nagarajan S., Maffulli N., Packkyarathinam R.P., Jeyaraman N., Nallakumarasamy A., Khanna M., Yadav S., Gupta A. (2023). Stem Cell Therapy in Critical Limb Ischemia. Cureus.

[112] Zhang X., Jiang Y., Huang Q., Wu Z., Pu H., Xu Z., Li B., Lu X., Yang X., Qin J. (2021). Exosomes derived from adipose-derived stem cells overexpressing glyoxalase-1 protect endothelial cells and enhance angiogenesis in type 2 diabetic mice with limb ischemia. Stem Cell Res. Ther..

[113] Lopatina T., Favaro E., Grange C., Cedrino M., Ranghino A., Occhipinti S., Fallo S., Buffolo F., Gaykalova D.A., Zanone M.M. (2018). PDGF enhances the protective effect of adipose stem cell-derived extracellular vesicles in a model of acute hindlimb ischemia. Sci. Rep..

[114] Qadura M., Terenzi D.C., Verma S., Al-Omran M., Hess D.A. (2018). Concise Review: Cell Therapy for Critical Limb Ischemia: An Integrated Review of Preclinical and Clinical Studies. Stem Cells.

[115] Dubský M., Husáková J., Sojáková D., Fejfarová V., Jude E.B. (2023). Cell Therapy of Severe Ischemia in People with Diabetic Foot Ulcers—Do We Have Enough Evidence?. Mol. Diagn. Ther..

[116] Panunzi A., Madotto F., Sangalli E., Riccio F., Sganzaroli A.B., Galenda P., Bertulessi A., Barmina M.F., Ludovico O., Fortunato O. (2022). Results of a prospective observational study of autologous peripheral blood mononuclear cell therapy for no-option critical limb-threatening ischemia and severe diabetic foot ulcers. Cardiovasc. Diabetol..

[117] Li M., Fang F., Sun M., Zhang Y., Hu M., Zhang J. (2022). Extracellular vesicles as bioactive nanotherapeutics: An emerging paradigm for regenerative medicine. Theranostics.

[118] Shirbaghaee Z., Keshel S.H., Rasouli M., Valizadeh M., Nazari S.S.H., Hassani M., Soleimani M. (2023). Report of a phase 1 clinical trial for safety assessment of human placental mesenchymal stem cells therapy in patients with critical limb ischemia (CLI). Stem Cell Res. Ther..

[119] Nelson B.C., Maragh S., Ghiran I.C., Jones J.C., DeRose P.C., Elsheikh E., Vreeland W.N., Wang L. (2020). Measurement and standardization challenges for extracellular vesicle therapeutic delivery vectors. Nanomedicine.

[120] Jin J.-F., Zhu L.-L., Chen M., Xu H.-M., Wang H.-F., Feng X.-Q., Zhu X.-P., Zhou Q. (2015). The optimal choice of medication administration route regarding intravenous, intramuscular, and subcutaneous injection. Patient Prefer. Adherence.

[121] Fuloria S., Subramaniyan V., Dahiya R., Dahiya S., Sudhakar K., Kumari U., Sathasivam K., Meenakshi D.U., Wu Y.S., Sekar M. (2021). Mesenchymal stem cell-derived extracellular vesicles: Regenerative potential and challenges. Biology.

[122] Poinsot V., Pizzinat N., Ong-Meang V. (2024). Engineered and Mimicked Extracellular Nanovesicles for Therapeutic Delivery. Nanomaterials.

[123] Gao J., Chu D., Wang Z. (2016). Cell Membrane-formed Nanovesicles for Disease-Targeted Delivery. J. Control. Release.

[124] Momin M.Y., Gaddam R.R., Kravitz M., Gupta A., Vikram A. (2021). The Challenges and Opportunities in the Development of MicroRNA Therapeutics: A Multidisciplinary Viewpoint. Cells.

[125] Rankin-Turner S., Vader P., O’Driscoll L., Giebel B., Heaney L.M., Davies O.G. (2021). A call for the standardised reporting of factors affecting the exogenous loading of extracellular vesicles with therapeutic cargos. Adv. Drug Deliv. Rev..

[126] Farzamfar S., Hasanpour A., Nazeri N., Razavi H., Salehi M., Shafei S., Nooshabadi V.T., Vaez A., Ehterami A., Sahrapeyma H. (2019). Extracellular micro/nanovesicles rescue kidney from ischemia-reperfusion injury. J. Cell. Physiol..

[127] de Jong O.G., Van Balkom B.W.M., Schiffelers R.M., Bouten C.V.C., Verhaar M.C. (2014). Extracellular Vesicles: Potential Roles in Regenerative Medicine. Front. Immunol..

[128] Sanz-Ros J., Mas-Bargues C., Romero-García N., Huete-Acevedo J., Dromant M., Borrás C. (2023). Extracellular Vesicles as Therapeutic Resources in the Clinical Environment. Int. J. Mol. Sci..

[129] Teraa M., Conte M.S., Moll F.L., Verhaar M.C. (2016). Critical limb ischemia: Current trends and future directions. J. Am. Heart Assoc..

[130] Klyachko N.L., Arzt C.J., Li S.M., Gololobova O.A., Batrakova E.V. (2020). Extracellular Vesicle-Based Therapeutics: Preclinical and Clinical Investigations. Pharmaceutics.

[131] Huang L., Wu E., Liao J., Wei Z., Wang J., Chen Z. (2023). Research Advances of Engineered Exosomes as Drug Delivery Carrier. ACS Omega.

[132] Ding S., Kim Y.-J., Huang K.-Y., Um D., Jung Y., Kong H. (2024). Delivery-mediated exosomal therapeutics in ischemia–reperfusion injury: Advances, mechanisms, and future directions. Nano Converg..

[133] Han C., Zhou J., Liu B., Liang C., Pan X., Zhang Y., Zhang Y., Wang Y., Shao L., Zhu B. (2019). Delivery of miR-675 by stem cell-derived exosomes encapsulated in silk fibroin hydrogel prevents aging-induced vascular dysfunction in mouse hindlimb. Mater. Sci. Eng. C Mater. Biol. Appl..

[134] Ungerleider J.L., Johnson T.D., Hernandez M.J., Elhag D.I., Braden R.L., Dzieciatkowska M., Osborn K.G., Hansen K.C., Mahmud E., Christman K.L. (2016). Extracellular Matrix Hydrogel Promotes Tissue Remodeling, Arteriogenesis, and Perfusion in a Rat Hindlimb Ischemia Model. JACC Basic Transl. Sci..

[135] Abdul-Rahman T., Roy P., Herrera-Calderón R.E., Khidri F.F., Omotesho Q.A., Rumide T.S., Fatima M., Roy S., Wireko A.A., Atallah O. (2024). Extracellular vesicle-mediated drug delivery in breast cancer theranostics. Discov. Oncol..

[136] Immunogenicity Testing of Therapeutic Protein Products—Developing and Validating Assays for Anti-Drug Antibody Detection|FDA. https://www.fda.gov/regulatory-information/search-fda-guidance-documents/immunogenicity-testing-therapeutic-protein-products-developing-and-validating-assays-anti-drug.

[137] Mondal J., Pillarisetti S., Junnuthula V., Surwase S.S., Hwang S.R., Park I.-K., Lee Y.-K. (2024). Extracellular vesicles and exosome-like nanovesicles as pioneering oral drug delivery systems. Front. Bioeng. Biotechnol..

[138] Lottes A.E., Cavanaugh K.J., Chan Y.Y.-F., Devlin V.J., Goergen C.J., Jean R., Linnes J.C., Malone M., Peat R., Reuter D.G. (2022). Navigating the Regulatory Pathway for Medical Devices—A Conversation with the FDA, Clinicians, Researchers, and Industry Experts. J. Cardiovasc. Transl. Res..

[139] Huang W., du Sert N.P., Vollert J., Rice A.S.C. (2020). General Principles of Preclinical Study Design. Handb. Exp. Pharmacol..

[140] Shen J., Swift B., Mamelok R., Pine S., Sinclair J., Attar M. (2019). Design and Conduct Considerations for First-in-Human Trials. Clin. Transl. Sci..

[141] McFadden E., Jackson J., Forrest J. (2022). Documentation: Essential Documents and Standard Operating Procedures. Princ. Pract. Clin. Trials.

[142] Yu J., Sane S., Kim J.-E., Yun S., Kim H.-J., Jo K.B., Wright J.P., Khoshdoozmasouleh N., Lee K., Oh H.T. (2023). Biogenesis and delivery of extracellular vesicles: Harnessing the power of EVs for diagnostics and therapeutics. Front. Mol. Biosci..

[143] Zhao C., Xing Z., Wei X., Liao G., Yang D., Liu H., Fan Y. (2023). Multibiofunctional TFEB-engineered endothelial progenitor cell-derived extracellular vesicles/hydrogel system for rescuing critical limb ischemia. Chem. Eng. J..

[144] Gangadaran P., Rajendran R.L., Lee H.W., Kalimuthu S., Hong C.M., Jeong S.Y., Lee S.-W., Lee J., Ahn B.-C. (2017). Extracellular vesicles from mesenchymal stem cells activates VEGF receptors and accelerates recovery of hindlimb ischemia. J. Control. Release.

[145] Doronzo G., Astanina E., Corà D., Chiabotto G., Comunanza V., Noghero A., Neri F., Puliafito A., Primo L., Spampanato C. (2019). TFEB controls vascular development by regulating the proliferation of endothelial cells. EMBO J..

[146] Mir B., Goettsch C. (2020). Extracellular Vesicles as Delivery Vehicles of Specific Cellular Cargo. Cells.

[147] Jin Y., Ma L., Zhang W., Yang W., Feng Q., Wang H. (2022). Extracellular signals regulate the biogenesis of extracellular vesicles. Biol. Res..

[148] Liu Y., Li Y., Zeng T. (2023). Multi-omics of extracellular vesicles: An integrative representation of functional mediators and perspectives on lung disease study. Front. Bioinform..

[149] Shaba E., Vantaggiato L., Governini L., Haxhiu A., Sebastiani G., Fignani D., Grieco G.E., Bergantini L., Bini L., Landi C. (2022). Multi-Omics Integrative Approach of Extracellular Vesicles: A Future Challenging Milestone. Proteomes.

[150] Chitoiu L., Dobranici A., Gherghiceanu M., Dinescu S., Costache M. (2020). Multi-Omics Data Integration in Extracellular Vesicle Biology—Utopia or Future Reality?. Int. J. Mol. Sci..

[151] Shahbazi R., Kalishwaralal K., Paul M.K., Anto R.J. (2023). Editorial: Role of extracellular vesicles (EVs) in pathogenesis, diagnosis, therapeutic delivery, treatment and theranostic applications in cancer. Front. Bioeng. Biotechnol..

[152] Mathur S., Sutton J. (2017). Personalized medicine could transform healthcare. Biomed. Rep..

[153] Bonilla O., Javier C. (2022). Extracellular Vesicles: A Novel Immunomodulator in Bladder Cancer Recurrence and BCG Immunotherapy. Ph.D. Thesis.

[154] Patel S.A., King C.C., Lim P.K., Habiba U., Dave M., Porecha R., Rameshwar P. (2010). Personalizing Stem Cell Research and Therapy: The Arduous Road Ahead or Missed Opportunity?. Curr. Pharmacogenomics Pers. Med..

[155] Goetz L.H., Schork N.J. (2018). Personalized Medicine: Motivation, Challenges and Progress. Fertil. Steril..

[156] Ranghino A., Cantaluppi V., Grange C., Vitillo L., Fop F., Biancone L., Deregibus M., Tetta C., Segoloni G., Camussi G. (2012). Endothelial progenitor cell-derived microvesicles improve neovascularization in a murine model of hindlimb ischemia. Int. J. Immunopathol. Pharmacol..

[157] Mathiyalagan P., Liang Y., Kim D., Misener S., Thorne T., Kamide C.E., Klyachko E., Losordo D.W., Hajjar R.J., Sahoo S. (2017). Angiogenic Mechanisms of Human CD34^+^ Stem Cell Exosomes in the Repair of Ischemic Hindlimb. Circ. Res..

[158] Qiu X., Zhou J., Xu Y., Liao L., Yang H., Xiang Y., Zhou Z., Sun Q., Chen M., Zhang J. (2022). Prophylactic exercise-derived circulating exosomal miR-125a-5p promotes endogenous revascularization after hindlimb ischemia by targeting endothelin converting enzyme 1. Front. Cardiovasc. Med..

[159] Hu G.-W., Li Q., Niu X., Hu B., Liu J., Zhou S.-M., Guo S.-C., Lang H.-L., Zhang C.-Q., Wang Y. (2015). Exosomes secreted by human-induced pluripotent stem cell-derived mesenchymal stem cells attenuate limb ischemia by promoting angiogenesis in mice. Stem Cell Res. Ther..

[160] Giraud R., Moyon A., Simoncini S., Duchez A.-C., Nail V., Chareyre C., Bouhlel A., Balasse L., Fernandez S., Vallier L. (2022). Tracking Radiolabeled Endothelial Microvesicles Predicts Their Therapeutic Efficacy: A Proof-of-Concept Study in Peripheral Ischemia Mouse Model Using SPECT/CT Imaging. Pharmaceutics.

[161] Lai R.C., Yeo R.W., Lim S.K. (2015). Mesenchymal stem cell exosomes. Semin. Cell Dev. Biol..

[162] Théry C., Zitvogel L., Amigorena S. (2002). Exosomes: Composition, biogenesis and function. Nat. Rev. Immunol..

[163] Vader P., Mol E.A., Pasterkamp G., Schiffelers R.M. (2016). Extracellular vesicles for drug delivery. Adv. Drug Deliv. Rev..

[164] Witwer K.W., Théry C. (2019). Extracellular vesicles or exosomes? On primacy, precision, and popularity influencing a choice of nomenclature. J. Extracell. Vesicles.

[165] Tkach M., Théry C. (2016). Communication by Extracellular Vesicles: Where We Are and Where We Need to Go. Cell.

[166] Robbins P.D., Morelli A.E. (2014). Regulation of immune responses by extracellular vesicles. Nat. Rev. Immunol..

[167] Ha D., Yang N., Nadithe V. (2016). Exosomes as therapeutic drug carriers and delivery vehicles across biological membranes: Current perspectives and future challenges. Acta Pharm. Sin. B.

[168] Sahoo S., Losordo D.W. (2014). Exosomes and cardiac repair after myocardial infarction. Circ. Res..

[169] Mathieu M., Martin-Jaular L., Lavieu G., Théry C. (2019). Specificities of secretion and uptake of exosomes and other extracellular vesicles for cell-to-cell communication. Nat. Cell Biol..

